# A Low-Complexity and Asymptotically Optimal Coding Strategy for Gaussian Vector Sources

**DOI:** 10.3390/e21100965

**Published:** 2019-10-02

**Authors:** Marta Zárraga-Rodríguez, Jesús Gutiérrez-Gutiérrez, Xabier Insausti

**Affiliations:** Tecnun, University of Navarra, Paseo de Manuel Lardizábal 13, 20018 San Sebastián, Spain; mzarraga@tecnun.es (M.Z.-R.); xinsausti@tecnun.es (X.I.)

**Keywords:** source coding, rate distortion function (RDF), Gaussian vector, asymptotically wide sense stationary (AWSS) vector source, block discrete Fourier transform (DFT)

## Abstract

In this paper, we present a low-complexity coding strategy to encode (compress) finite-length data blocks of Gaussian vector sources. We show that for large enough data blocks of a Gaussian asymptotically wide sense stationary (AWSS) vector source, the rate of the coding strategy tends to the lowest possible rate. Besides being a low-complexity strategy it does not require the knowledge of the correlation matrix of such data blocks. We also show that this coding strategy is appropriate to encode the most relevant Gaussian vector sources, namely, wide sense stationary (WSS), moving average (MA), autoregressive (AR), and ARMA vector sources.

## 1. Introduction

The rate distortion function (RDF) of a source provides the minimum rate at which data can be encoded in order to be able to recover them with a mean squared error (MSE) per dimension not larger than a given distortion.

In this paper, we present a low-complexity coding strategy to encode (compress) finite-length data blocks of Gaussian *N*-dimensional vector sources. Moreover, we show that for large enough data blocks of a Gaussian asymptotically wide sense stationary (AWSS) vector source, the rate of our coding strategy tends to the RDF of the source. The definition of AWSS vector process can be found in ([1] (Definition 7.1)). This definition was first introduced for the scalar case N = 1 (see ([2] (Section 6)) or [3]), and it is based on the Gray concept of asymptotically equivalent sequences of matrices [4].

A low-complexity coding strategy can be found in [5] for finite-length data blocks of Gaussian wide sense stationary (WSS) sources and in [6] for finite-length data blocks of Gaussian AWSS autoregressive (AR) sources. Both precedents deal with scalar processes. The low-complexity coding strategy presented in this paper generalizes the aforementioned strategies to Gaussian AWSS vector sources.

Our coding strategy is based on the block discrete Fourier transform (DFT), and therefore, it turns out to be a low-complexity coding strategy when the fast Fourier transform (FFT) algorithm is used. Specifically, the computational complexity of our coding strategy is O(nNlogn), where *n* is the length of the data blocks. Besides being a low-complexity strategy, it does not require the knowledge of the correlation matrix of such data blocks.

We show that this coding strategy is appropriate to encode the most relevant Gaussian vector sources, namely, WSS, moving average (MA), autoregressive (AR), and ARMA vector sources. Observe that our coding strategy is then appropriate to encode Gaussian vector sources found in the literature, such as the corrupted WSS vector sources considered in [7,8] for the quadratic Gaussian CEO problem.

The paper is organized as follows. In Section 2, we obtain several new mathematical results on the block DFT, and we present an upper bound for the RDF of a complex Gaussian vector. In Section 3, using the results given in Section 2, we present a new coding strategy based on the block DFT to encode finite-length data blocks of Gaussian vector sources. In Section 4, we show that for large enough data blocks of a Gaussian AWSS vector source, the rate of our coding strategy tends to the RDF of the source. In Section 5, we show that our coding strategy is appropriate to encode WSS, MA, AR, and ARMA vector sources. In Section 6, conclusions and numerical examples are presented.

## 2. Preliminaries

### 2.1. Notation

In this paper N, Z, R, and C are the set of positive integers, the set of integers, the set of real numbers, and the set of complex numbers, respectively. The symbol ⊤ denotes transpose and the symbol ∗ denotes conjugate transpose. ${∥\cdot ∥}_{2}$ and ${∥\cdot ∥}_{F}$ are the spectral and the Frobenius norm, respectively. ⌈x⌉ denotes the smallest integer higher than or equal to *x*. *E* stands for expectation, ⊗ is the Kronecker product, and $\lambda_{j}\left(A\right)$, j∈{1,…,n}, denote the eigenvalues of an n×n Hermitian matrix *A* arranged in decreasing order. $R^{n\times 1}$ is the set of real *n*-dimensional (column) vectors, $C^{m\times n}$ denotes the set of m×n complex matrices, $0_{m\times n}$ is the m×n zero matrix, $I_{n}$ denotes the n×n identity matrix, and $V_{n}$ is the n×n Fourier unitary matrix, i.e.,

$${\left[V_{n}\right]}_{j,k}=\frac{1}{\sqrt{n}}e^{-\frac{2\pi (j-1)(k-1)}{n}i},\;j,k\in \left\{1,\ldots ,n\right\},$$

where i is the imaginary unit.

If $A_{j}\in C^{N\times N}$ for all j∈{1,…,n}, then ${\mathrm{diag}}_{1\leq j\leq n}\left(A_{j}\right)$ denotes the n×n block diagonal matrix with N×N blocks given by ${\mathrm{diag}}_{1\leq j\leq n}\left(A_{j}\right)={\left(A_{j}\delta_{j,k}\right)}_{j,k=1}^{n}$, where δ is the Kronecker delta.

Re and Im denote the real part and the imaginary part of a complex number, respectively. If $A\in C^{m\times n}$, then Re(A) and Im(A) are the m×n real matrices given by [Re(A)]j,k=Re([A]j,k) and [Im(A)]j,k=Im([A]j,k) with j∈{1,…,m} and k∈{1,…,n}, respectively.

If $z\in C^{N\times 1}$, then $\hat{z}$ denotes the real 2N-dimensional vector given by

z^=Re(z)Im(z).

If $z_{k}\in C^{N\times 1}$ for all k∈{1,…,n}, then $z_{n:1}$ is the nN-dimensional vector given by

$$z_{n:1}=\left(\begin{array}{c} z_{n}\\ z_{n-1}\\ \vdots \\ z_{1} \end{array}\right).$$

Finally, if $z_{k}$ is a (complex) random *N*-dimensional vector for all k∈N, $\left\{z_{k}\right\}$ denotes the corresponding (complex) random *N*-dimensional vector process.

### 2.2. New Mathematical Results on the Block DFT

We first give a simple result on the block DFT of real vectors.

**Lemma** **1.** *Let n,N∈N. Consider $x_{k}\in C^{N\times 1}$ for all k∈{1,…,n}. Suppose that $y_{n:1}$ is the block DFT of $x_{n:1}$, i.e.,*

(1) $$y_{n:1}=\left(V_{n}^{*}⊗I_{N}\right)x_{n:1}={\left(V_{n}⊗I_{N}\right)}^{*}x_{n:1}.$$

*Then the two following assertions are equivalent:*

*1.*  *$x_{n:1}\in R^{nN\times 1}$.*
*2.*  *$y_{k}=\bar{y_{n-k}}$ for all k∈{1,…,n−1} and $y_{n}\in R^{N\times 1}$.*

**Proof.**  See Appendix A. □

We now give three new mathematical results on the block DFT of random vectors that are used in Section 3.

**Theorem** **1.** *Consider n,N∈N. Let $x_{k}$ be a random N-dimensional vector for all k∈{1,…,n}. Suppose that $y_{n:1}$ is given by Equation (1). If k∈{1,…,n}, then*

(2) $$\lambda_{nN}\left(E\left(x_{n:1}x_{n:1}^{*}\right)\right)\leq \lambda_{N}\left(E(x_{k}x_{k}^{*})\right)\leq \lambda_{1}\left(E(x_{k}x_{k}^{*})\right)\leq \lambda_{1}\left(E\left(x_{n:1}x_{n:1}^{*}\right)\right)$$

*and*

(3) $$\lambda_{nN}\left(E\left(x_{n:1}x_{n:1}^{*}\right)\right)\leq \lambda_{N}\left(E(y_{k}y_{k}^{*})\right)\leq \lambda_{1}\left(E(y_{k}y_{k}^{*})\right)\leq \lambda_{1}\left(E\left(x_{n:1}x_{n:1}^{*}\right)\right).$$

**Proof.**  See Appendix B. □

**Theorem** **2.** *Let $x_{n:1}$ and $y_{n:1}$ be as in Theorem 1. Suppose that $x_{n:1}$ is real. If $k\in \left\{1,\ldots ,n-1\right\}\setminus \left\{\frac{n}{2}\right\}$, then*

$$\frac{\lambda_{nN}\left(E\left(x_{n:1}x_{n:1}^{⊤}\right)\right)}{2}\leq \lambda_{2N}\left(E\left(\hat{y_{k}}{\hat{y_{k}}}^{⊤}\right)\right)\leq \lambda_{1}\left(E\left(\hat{y_{k}}{\hat{y_{k}}}^{⊤}\right)\right)\leq \frac{\lambda_{1}\left(E\left(x_{n:1}x_{n:1}^{⊤}\right)\right)}{2}.$$

**Proof.**  See Appendix C. □

**Lemma** **2.** *Let $x_{n:1}$ and $y_{n:1}$ be as in Theorem 1. If k∈{1,…,n}, then*

*1.*  $E\left(y_{k}y_{k}^{*}\right)={\left[{\left(V_{n}⊗I_{N}\right)}^{*}E\left(x_{n:1}x_{n:1}^{*}\right)\left(V_{n}⊗I_{N}\right)\right]}_{n-k+1,n-k+1}$.
*2.*  $E\left(y_{k}y_{k}^{⊤}\right)={\left[{\left(V_{n}⊗I_{N}\right)}^{*}E\left(x_{n:1}x_{n:1}^{⊤}\right)\left(\bar{V_{n}⊗I_{N}}\right)\right]}_{n-k+1,n-k+1}$.
*3.*  Eyk^yk^⊤=12ReEykyk*+ReEykyk⊤ImEykyk⊤−ImEykyk*ImEykyk*+ImEykyk⊤ReEykyk*−ReEykyk⊤.

**Proof.**  See Appendix D. □

### 2.3. Upper Bound for the RDF of a Complex Gaussian Vector

In [9], Kolmogorov gave a formula for the RDF of a real zero-mean Gaussian *N*-dimensional vector x with positive definite correlation matrix $E\left(xx^{⊤}\right)$, namely,

(4) $$R_{x}\left(D\right)=\frac{1}{N}\sum_{k=1}^{N}\max\left\{0,\frac{1}{2}\ln\frac{\lambda_{k}\left(E\left(xx^{⊤}\right)\right)}{\theta }\right\}\;\forall D\in \left(0,\frac{\mathrm{tr}\left(E\left(xx^{⊤}\right)\right)}{N}\right],$$

where tr denotes trace and θ is a real number satisfying

$$D=\frac{1}{N}\sum_{k=1}^{N}\min\left\{\theta ,\lambda_{k}\left(E\left(xx^{⊤}\right)\right)\right\}.$$

If $D\in \left(0,\lambda_{N}\left(E\left(xx^{⊤}\right)\right)\right]$, an optimal coding strategy to achieve $R_{x}\left(D\right)$ is to encode ${\left[z\right]}_{1,1},\ldots ,{\left[z\right]}_{N,1}$ separately, where $z=U^{⊤}x$ with *U* being a real orthogonal eigenvector matrix of $E\left(xx^{⊤}\right)$ (see ([6] (Corollary 1))). Observe that in order to obtain *U*, we need to know the correlation matrix $E\left(xx^{⊤}\right)$. This coding strategy also requires an optimal coding method for real Gaussian random variables. Moreover, as $0<D\leq \lambda_{N}\left(E\left(xx^{⊤}\right)\right)\leq \frac{1}{N}\sum_{k=1}^{N}\lambda_{k}\left(E\left(xx^{⊤}\right)\right)=\frac{\mathrm{tr}\left(E\left(xx^{⊤}\right)\right)}{N}$, if $D\in \left(0,\lambda_{N}\left(E\left(xx^{⊤}\right)\right)\right]$, then from Equation (4) we obtain

(5) $$R_{x}\left(D\right)=\frac{1}{N}\sum_{k=1}^{N}\frac{1}{2}\ln\frac{\lambda_{k}\left(E\left(xx^{⊤}\;\right)\right)}{D}=\frac{1}{2N}\ln\frac{\prod_{k=1}^{N}\lambda_{k}\left(E\left(xx^{⊤}\;\right)\right)}{D^{N}}=\frac{1}{2N}\ln\frac{\det\left(E\left(xx^{⊤}\right)\right)}{D^{N}}.$$

We recall that $R_{x}\left(D\right)$ can be thought of as the minimum rate (measured in nats) at which x can be encoded (compressed) in order to be able to recover it with an MSE per dimension not larger than *D*, that is:

$$\frac{E\left({∥x-\tilde{x}∥}_{2}^{2}\right)}{N}\leq D,$$

where $\tilde{x}$ denotes the estimation of x.

The following result gives an upper bound for the RDF of a complex zero-mean Gaussian *N*-dimensional vector (i.e., a real zero-mean Gaussian 2N-dimensional vector).

**Lemma** **3.** *Consider N∈N. Let z be a complex zero-mean Gaussian N-dimensional vector. If $E\left(\hat{z}\;\hat{z}{\;}^{⊤}\right)$ is a positive definite matrix, then*

(6) $$R_{\hat{z}}\left(D\right)\leq \frac{1}{2N}\ln\frac{\det\left(E\left(zz^{*}\right)\right)}{{\left(2D\right)}^{N}}\;\forall D\in \left(0,\lambda_{2N}\left(E\left(\hat{z}\;\hat{z}{\;}^{⊤}\right)\right)\right].$$

**Proof.**  We divide the proof into three steps:

*Step 1:* We prove that $E\left(zz^{*}\right)$ is a positive definite matrix. We have

Ez^z^⊤=ERe(z)Re(z)⊤ERe(z)Im(z)⊤EIm(z)Re(z)⊤EIm(z)Im(z)⊤

and

Ezz*=ERe(z)+iIm(z)Re(z)⊤−iIm(z)⊤=ERe(z)Re(z)⊤+EIm(z)Im(z)⊤+iEIm(z)Re(z)⊤−iERe(z)Im(z)⊤.

Consider $u\in C^{N\times 1}$, and suppose that $u^{*}E\left(zz^{*}\right)u=0$. We only need to show that $u=0_{N\times 1}$. As $E\left(\hat{z}\;\hat{z}{\;}^{⊤}\right)$ is a positive definite matrix and

u−iu*Ez^z^⊤u−iu=u−iu*ERe(z)Re(z)⊤u−iERe(z)Im(z)⊤uEIm(z)Re(z)⊤u−iEIm(z)Im(z)⊤u=u*ERe(z)Re(z)⊤u−iu*ERe(z)Im(z)⊤u+iu*EIm(z)Re(z)⊤u+u*EIm(z)Im(z)⊤u=u*Ezz*u=0,

we obtain $\left(\;\;\;\begin{array}{c} u\\ -iu \end{array}\;\;\;\right)=0_{2N\times 1}$, or equivalently $u=0_{N\times 1}$.

*Step 2:* We show that $\det\left(\;E\;\left(\hat{z}\;\hat{z}{\;}^{⊤}\right)\right)\leq \frac{{\left(\det\left(E\left(zz^{*}\right)\right)\right)}^{2}}{2^{2N}}$. We have $\;E\;\left(zz^{*}\right)=\;\Lambda_{c}\;+i\Lambda_{s}$, where Λc=ERe(z)Re(z)⊤+EIm(z)Im(z)⊤ and Λs=EIm(z)Re(z)⊤−EIm(z)Re(z)⊤⊤. Applying ([10] (Corollary 1)), we obtain

$$\begin{array}{cc} \det\left(E\left(\hat{z}\;\hat{z}{\;}^{⊤}\right)\right)\; & \leq \frac{\det\left(\Lambda_{c}+\Lambda_{s}\Lambda_{c}^{-1}\Lambda_{s}\right)\det\left(\Lambda_{c}\right)}{2^{2N}}=\frac{\det\left(I_{N}+\Lambda_{s}\Lambda_{c}^{-1}\Lambda_{s}\Lambda_{c}^{-1}\right){\left(\det\left(\Lambda_{c}\right)\right)}^{2}}{2^{2N}}\\ & =\frac{\det\left(\left(I_{N}+i\Lambda_{s}\Lambda_{c}^{-1}\right)\left(I_{N}-i\Lambda_{s}\Lambda_{c}^{-1}\right)\right){\left(\det\left(\Lambda_{c}\right)\right)}^{2}}{2^{2N}}=\frac{\det\left(\Lambda_{c}+i\Lambda_{s}\right)\det\left(\Lambda_{c}-i\Lambda_{s}\right)}{2^{2N}}\\ & =\frac{\det\left(E\left(zz^{*}\right)\right)\det\left(\bar{E\left(zz^{*}\right)}\right)}{2^{2N}}=\frac{\det\left(E\left(zz^{*}\right)\right)\bar{\det\left(E\left(zz^{*}\right)\right)}}{2^{2N}}=\frac{{\left(\det\left(E\left(zz^{*}\right)\right)\right)}^{2}}{2^{2N}}. \end{array}$$

*Step 3:* We now prove Equation (6). From Equation (5), we conclude that

$$\begin{array}{c} R_{\hat{z}}\left(D\right)=\frac{1}{4N}\ln\frac{\det\left(E\left(\hat{z}\;\hat{z}{\;}^{⊤}\right)\;\right)}{D^{2N}}\leq \frac{1}{4N}\ln\frac{{\left(\det\left(E\left(zz^{*}\right)\right)\right)}^{2}}{{\left(2D\right)}^{2N}}=\frac{1}{2N}\ln\frac{\det\left(E\left(zz^{*}\right)\right)}{{\left(2D\right)}^{N}}. \end{array}$$

 □

## 3. Low-Complexity Coding Strategy for Gaussian Vector Sources

**Figure 1:**
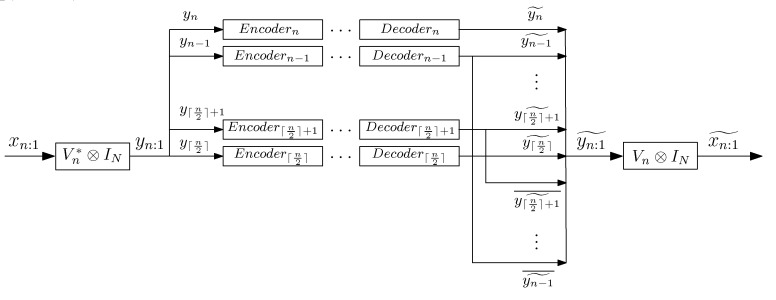
Proposed coding strategy for Gaussian vector sources. In this figure, $Encoder_{k}$ ($Decoder_{k}$) denotes the optimal encoder (decoder) for the Gaussian *N*-dimensional vector $y_{k}$ with $k\in \left\{\left\lceil \frac{n}{2}\right\rceil ,\ldots ,n\right\}$.

In this section (see Theorem 3), we present our coding strategy for Gaussian vector sources. To encode a finite-length data block $x_{n:1}$ of a Gaussian *N*-dimensional vector source $\left\{x_{k}\right\}$, we compute the block DFT of $x_{n:1}$ ($y_{n:1}$) and we encode $y_{\left\lceil \frac{n}{2}\right\rceil },\ldots ,y_{n}$ separately with $\frac{E\left({∥y_{k}-\tilde{y_{k}}∥}_{2}^{2}\right)}{N}\leq D$ for all $k\in \left\{\left\lceil \frac{n}{2}\right\rceil ,\ldots ,n\right\}$ (see Figure 1).

We denote by ${\tilde{R}}_{x_{n:1}}\left(D\right)$ the rate of our strategy. Theorem 3 also provides an upper bound of ${\tilde{R}}_{x_{n:1}}\left(D\right)$. This upper bound is used in Section 4 to prove that our coding strategy is asymptotically optimal whenever the Gaussian vector source is AWSS.

In Theorem 3 $C_{A_{n}}$ denotes the matrix $\left(V_{n}\;⊗\;I_{N}\right){\mathrm{diag}}_{1\leq k\leq n}\left({\left[{\left(V_{n}\;⊗\;I_{N}\right)}^{*}A_{n}\left(V_{n}\;⊗\;I_{N}\right)\right]}_{k,k}\right){\left(V_{n}\;⊗\;I_{N}\right)}^{*}$, where $A_{n}\in C^{nN\times nN}$.

**Theorem** **3.** *Consider n,N∈N. Let $x_{k}$ be a random N-dimensional vector for all k∈{1,…,n}. Suppose that $x_{n:1}$ is a real zero-mean Gaussian vector with a positive definite correlation matrix (or equivalently, $\lambda_{nN}\left(E\left(x_{n:1}x_{n:1}^{⊤}\right)\right)>0$). Let $y_{n:1}$ be the random vector given by Equation (1). If $D\in \left(0,\lambda_{nN}\left(E\left(x_{n:1}x_{n:1}^{⊤}\right)\right)\right]$, then*

(7) $$\begin{array}{cc} R_{x_{n:1}} & \left(D\right)\leq {\tilde{R}}_{x_{n:1}}\left(D\right)\;\leq \;\frac{1}{2nN}\ln\frac{\det\left(C_{E\left(x_{n:1}x_{n:1}^{⊤}\right)}\right)}{D^{nN}}, \end{array}$$

*where*

$${\tilde{R}}_{x_{n:1}}\left(D\right)=\left\{\begin{array}{cc} \frac{R_{y_{\frac{n}{2}}}\left(D\right)+2\sum_{k=\frac{n}{2}+1}^{n-1}R_{\hat{y_{k}}}\left(\frac{D}{2}\right)+R_{y_{n}}\left(D\right)}{n} & if\;n\;is\;even,\;\\ \frac{2\sum_{k=\frac{n+1}{2}}^{n-1}R_{\hat{y_{k}}}\left(\frac{D}{2}\right)+R_{y_{n}}\left(D\right)}{n} & if\;n\;is\;odd. \end{array}\right.$$

*Moreover,*

(8) $$\begin{array}{c} 0\leq \frac{1}{2nN}\ln\frac{\det\left(C_{E\left(x_{n:1}x_{n:1}^{⊤}\right)}\right)}{D^{nN}}-R_{x_{n:1}}\left(D\right)\leq \frac{1}{2}\ln\left(1+\frac{{∥E\left(x_{n:1}x_{n:1}^{⊤}\right)-C_{E\left(x_{n:1}x_{n:1}^{⊤}\right)}∥}_{F}}{\sqrt{nN}\lambda_{nN}\left(E\left(x_{n:1}x_{n:1}^{⊤}\right)\right)}\right). \end{array}$$

**Proof.**  We divide the proof into three steps:

*Step 1:* We show that $R_{x_{n:1}}\left(D\right)\leq {\tilde{R}}_{x_{n:1}}\left(D\right)$. From Lemma 1, $y_{k}=\bar{y_{n-k}}$ for all $k\in \{1,\ldots ,\left\lceil \frac{n}{2}\right\rceil -1\}$, and $y_{k}\in R^{N\times 1}$ with $k\in \{\frac{n}{2},n\}\cap N$. We encode $y_{\left\lceil \frac{n}{2}\right\rceil },\ldots ,y_{n}$ separately (i.e., if *n* is even, we encode $y_{\frac{n}{2}},\hat{y_{\frac{n}{2}+1}},\ldots ,\hat{y_{n-1}},y_{n}$ separately, and if *n* is odd, we encode $\hat{y_{\frac{n+1}{2}}},\ldots ,\hat{y_{n-1}},y_{n}$ separately) with

$$\frac{E\left({∥\hat{y_{k}}-\tilde{\hat{y_{k}}}∥}_{2}^{2}\right)}{2N}\leq \frac{D}{2},\;k\in \left\{\left\lceil \frac{n}{2}\right\rceil \ldots ,n-1\right\}\setminus \left\{\frac{n}{2}\right\}$$

and

$$\frac{E\left({∥y_{k}-\tilde{y_{k}}∥}_{2}^{2}\right)}{N}\leq D,\;k\in \left\{\frac{n}{2},n\right\}\cap N.$$

Let $\tilde{x_{n:1}}=\left(V_{n}⊗I_{N}\right)\tilde{y_{n:1}}$ with

$$\tilde{y_{n:1}}=\left(\begin{array}{c} \tilde{y_{n}}\\ \vdots \\ \tilde{y_{1}} \end{array}\right),$$

where $\hat{\tilde{y_{k}}}=\tilde{\hat{y_{k}}}$ for all $k\in \left\{\left\lceil \frac{n}{2}\right\rceil \ldots ,n-1\right\}\setminus \left\{\frac{n}{2}\right\}$, and $\tilde{y_{k}}=\bar{\tilde{y_{n-k}}}$ for all $k\in \{1,\ldots ,\left\lceil \frac{n}{2}\right\rceil -1\}$. Applying Lemma 1 yields $\tilde{x_{n:1}}\in R^{nN\times 1}$. As ${\left(V_{n}⊗I_{N}\right)}^{*}$ is unitary and ${∥\cdot ∥}_{2}$ is unitarily invariant, we have

$$\begin{array}{cc} \frac{E\left({∥x_{n:1}-\tilde{x_{n:1}}∥}_{2}^{2}\right)}{nN}\; & =\;\frac{E\left({∥{\left(V_{n}⊗I_{N}\right)}^{*}x_{n:1}-{\left(V_{n}⊗I_{N}\right)}^{*}\tilde{x_{n:1}}∥}_{2}^{2}\right)}{nN}\;\\ & =\;\frac{E\left({∥y_{n:1}-\tilde{y_{n:1}}∥}_{2}^{2}\right)}{nN}\;=\;\frac{1}{nN}\sum_{k=1}^{n}E\left({∥y_{k}-\tilde{y_{k}}∥}_{2}^{2}\right)\;\\ & =\;\frac{1}{nN}\left(2\sum_{k_{1}\in \left\{\left\lceil \frac{n}{2}\right\rceil \ldots ,n-1\right\}\setminus \left\{\frac{n}{2}\right\}}E\left({∥y_{k_{1}}-\tilde{y_{k_{1}}}∥}_{2}^{2}\right)\;+\sum_{k_{2}\in \left\{\frac{n}{2},n\right\}\cap N}E\left({∥y_{k_{2}}-\tilde{y_{k_{2}}}∥}_{2}^{2}\right)\;\right)\\ & =\;\frac{1}{nN}\left(2\sum_{k_{1}\in \left\{\left\lceil \frac{n}{2}\right\rceil \ldots ,n-1\right\}\setminus \left\{\frac{n}{2}\right\}}E\left({∥\hat{y_{k_{1}}}-\tilde{\hat{y_{k_{1}}}}∥}_{2}^{2}\right)\;+\sum_{k_{2}\in \left\{\frac{n}{2},n\right\}\cap N}E\left({∥y_{k_{2}}-\tilde{y_{k_{2}}}∥}_{2}^{2}\right)\;\right)\\ & \leq \;\left\{\begin{array}{cc} \;\frac{1}{nN}\left(2\left(\frac{n}{2}-1\right)ND+2ND\right) & \mathrm{if}n\mathrm{is}\mathrm{even},\\ \;\frac{1}{nN}\left(2\left(n-\frac{n+1}{2}\right)ND+ND\right) & \mathrm{if}n\mathrm{is}\mathrm{odd}, \end{array}\right\}=D. \end{array}$$

Consequently,

$$R_{x_{n:1}}\left(D\right)\leq \left\{\begin{array}{cc} \frac{NR_{y_{\frac{n}{2}}}\left(D\right)+2N\sum_{k=\frac{n}{2}+1}^{n-1}R_{\hat{y_{k}}}\left(\frac{D}{2}\right)+NR_{y_{n}}\left(D\right)}{nN} & \mathrm{if}n\mathrm{is}\mathrm{even},\;\\ \frac{2N\sum_{k=\frac{n+1}{2}}^{n-1}R_{\hat{y_{k}}}\left(\frac{D}{2}\right)+NR_{y_{n}}\left(D\right)}{nN} & \mathrm{if}n\mathrm{is}\mathrm{odd}, \end{array}\right\}={\tilde{R}}_{x_{n:1}}\left(D\right).$$

*Step 2:* We prove that ${\tilde{R}}_{x_{n:1}}\left(D\right)\leq \frac{1}{2nN}\ln\frac{\det\left(C_{E\;\left(x_{n:1}x_{n:1}^{⊤}\right)}\right)}{D^{nN}}$. From Equations (3) and (5), we obtain

(9) $$R_{y_{k}}\left(D\right)=\frac{1}{2N}\ln\frac{\det\left(E\left(y_{k}y_{k}^{⊤}\right)\right)}{D^{N}},\;k\in \left\{\frac{n}{2},n\right\}\cap N,$$

and applying Theorem 2 and Equation (5) yields

(10) $$R_{\hat{y_{k}}}\left(\frac{D}{2}\right)=\frac{1}{4N}\ln\frac{\det\left(E\left(\hat{y_{k}}{\hat{y_{k}}}^{⊤}\right)\right)}{{\left(\frac{D}{2}\right)}^{2N}},\;k\in \left\{1,\ldots ,n-1\right\}\setminus \left\{\frac{n}{2}\right\}.$$

From Lemma 3, we have

$$\begin{array}{cc} \; & {\tilde{R}}_{x_{n:1}}\left(D\right)\\ \; & \leq \frac{1}{n}\;\left[2\;\sum_{k_{1}\in \left\{\left\lceil \frac{n}{2}\right\rceil ,\ldots ,n-1\right\}\setminus \left\{\frac{n}{2}\right\}}\;\frac{1}{2N}\ln\frac{\det\left(\;E\;\left(y_{k_{1}}y_{k_{1}}^{*}\right)\right)}{D^{N}}\;+\;\sum_{k_{2}\in \left\{\frac{n}{2},n\right\}\cap N}\;\frac{1}{2N}\ln\frac{\det\left(\;E\;\left(y_{k_{2}}y_{k_{2}}^{*}\right)\right)}{D^{N}}\right]\\ \; & =\frac{1}{2nN}\;\left[\;\sum_{k_{1}\in \left\{\left\lceil \frac{n}{2}\right\rceil ,\ldots ,n-1\right\}\setminus \;\left\{\frac{n}{2}\right\}}\;\;\left(\;\;\ln\frac{\det\left(\;E\;\left(y_{k_{1}}y_{k_{1}}^{*}\right)\;\right)}{D^{N}}+\ln\frac{\bar{\det\left(\;E\;\left(y_{k_{1}}y_{k_{1}}^{*}\right)\;\right)}}{D^{N}}\;\;\right)\;\;\;+\;\sum_{k_{2}\in \left\{\frac{n}{2},n\right\}\cap N}\;\ln\frac{\det\left(\;E\;\left(y_{k_{2}}y_{k_{2}}^{*}\right)\;\right)}{D^{N}}\right]\\ \; & =\frac{1}{2nN}\;\left[\;\sum_{k_{1}\in \left\{\left\lceil \frac{n}{2}\right\rceil ,\ldots ,n-1\right\}\setminus \left\{\frac{n}{2}\right\}}\;\;\left(\;\;\ln\frac{\det\left(\;E\;\left(y_{k_{1}}y_{k_{1}}^{*}\right)\right)}{D^{N}}+\ln\frac{\det\left(\;E\;\left(y_{n-k_{1}}y_{n-k_{1}}^{*}\right)\right)}{D^{N}}\;\;\right)\;\;\;\right.\\ \; & \left.\;\;\;\;\;\;+\;\sum_{k_{2}\in \left\{\frac{n}{2},n\right\}\cap N}\;\ln\frac{\det\left(\;E\;\left(y_{k_{2}}y_{k_{2}}^{*}\right)\right)}{D^{N}}\right]\\ \; & =\frac{1}{2nN}\sum_{k=1}^{n}\ln\frac{\det\left(E\left(y_{k}y_{k}^{*}\right)\right)}{D^{N}}=\frac{1}{2nN}\ln\frac{\prod_{k=1}^{n}\det\left(E\left(y_{k}y_{k}^{*}\right)\right)}{D^{nN}}. \end{array}$$

As

(11) $$\begin{array}{cc} \; & \left\{\lambda_{j}\left(E\left(y_{k}y_{k}^{*}\right)\right):j\in \left\{1,\ldots ,N\right\},k\in \left\{1,\ldots ,n\right\}\right\}=\left\{\lambda_{j}\left({\left[E\left(y_{n:1}y_{n:1}^{*}\right)\right]}_{k,k}\right):j\in \left\{1,\ldots ,N\right\},k\in \left\{1,\ldots ,n\right\}\right\}\\ \; & \;\;\;\;\;\;\;\;=\left\{\lambda_{j}\left({\left[\;\left(\;V_{n}\;⊗\;I_{N}\;\right){\;}^{*}E\;\left(x_{n:1}x_{n:1}^{⊤}\right)\;\;\left(\;V_{n}\;⊗\;I_{N}\;\right)\;\right]}_{k,k}\right):j\in \left\{1,\ldots ,N\right\},k\in \left\{1,\ldots ,n\right\}\right\}\\ \; & \;\;\;\;\;\;\;\;=\left\{\lambda_{j}\left({\mathrm{diag}}_{1\leq k\leq n}\left({\left[\;\left(\;V_{n}\;⊗\;I_{N}\;\right){\;}^{*}E\;\left(x_{n:1}x_{n:1}^{⊤}\right)\;\;\left(\;V_{n}\;⊗\;I_{N}\;\right)\;\right]}_{k,k}\right)\right):j\in \left\{1,\ldots ,nN\right\}\right\}\\ \; & \;\;\;\;\;\;\;\;=\left\{\lambda_{j}\left(\left(\;V_{n}\;⊗\;I_{N}\;\right){\mathrm{diag}}_{1\leq k\leq n}\left({\left[\;\left(\;V_{n}\;⊗\;I_{N}\;\right){\;}^{*}E\;\left(x_{n:1}x_{n:1}^{⊤}\right)\;\;\left(\;V_{n}\;⊗\;I_{N}\;\right)\;\right]}_{k,k}\right){\left(\;V_{n}\;⊗\;I_{N}\;\right)}^{-1}\right):j\in \left\{1,\ldots ,nN\right\}\right\}\\ \; & \;\;\;\;\;\;\;\;=\left\{\lambda_{j}\left(C_{E\left(x_{n:1}x_{n:1}^{⊤}\right)}\right):j\in \left\{1,\ldots ,nN\right\}\right\}, \end{array}$$

we obtain

$$\begin{array}{c} \prod_{k=1}^{n}\det\left(E\left(y_{k}y_{k}^{*}\right)\right)=\prod_{k=1}^{n}\prod_{j=1}^{N}\lambda_{j}\left(E\left(y_{k}y_{k}^{*}\right)\right)=\prod_{j=1}^{nN}\lambda_{j}\left(C_{E\left(x_{n:1}x_{n:1}^{⊤}\right)}\right)=\det\left(C_{E\left(x_{n:1}x_{n:1}^{⊤}\right)}\right). \end{array}$$

*Step 3:* We show Equation (8).

As $E\left(x_{n:1}x_{n:1}^{⊤}\right)$ is a positive definite matrix (or equivalently, $E\left(x_{n:1}x_{n:1}^{⊤}\right)$ is Hermitian and $\lambda_{j}\left(E\left(x_{n:1}x_{n:1}^{⊤}\right)\right)>0$ for all j∈{1,…,nN}), ${\left(\;V_{n}\;⊗\;I_{N}\;\right)}^{*}E\left(x_{n:1}x_{n:1}^{⊤}\right)\left(\;V_{n}\;⊗\;I_{N}\;\right)$ is Hermitian. Hence, ${\left[{\left(\;V_{n}\;⊗\;I_{N}\;\right)}^{*}E\left(x_{n:1}x_{n:1}^{⊤}\right)\left(\;V_{n}\;⊗\;I_{N}\;\right)\right]}_{k,k}$ is Hermitian for all k∈{1,…,n}, and therefore, ${\mathrm{diag}}_{1\leq k\leq n}\left({\left[{\left(\;V_{n}\;⊗\;I_{N}\;\right)}^{*}E\left(x_{n:1}x_{n:1}^{⊤}\right)\left(\;V_{n}\;⊗\;I_{N}\;\right)\right]}_{k,k}\right)$ is also Hermitian. Consequently, $\left(\;V_{n}\;⊗\;I_{N}\;\right){\mathrm{diag}}_{1\leq k\leq n}\left({\left[{\left(\;V_{n}\;⊗\;I_{N}\;\right)}^{*}E\left(x_{n:1}x_{n:1}^{⊤}\right)\left(\;V_{n}\;⊗\;I_{N}\;\right)\right]}_{k,k}\right){\left(\;V_{n}\;⊗\;I_{N}\;\right)}^{*}$ is Hermitian, and applying Equations (3) and (11), we have that $C_{E\left(x_{n:1}x_{n:1}^{⊤}\right)}$ is a positive definite matrix.

Let $E\left(x_{n:1}x_{n:1}^{⊤}\right)=U{\mathrm{diag}}_{1\leq j\leq nN}\;\;\left(\lambda_{j}\left(E\left(x_{n:1}x_{n:1}^{⊤}\right)\right)\right)U^{-1}$ be an eigenvalue decomposition (EVD) of $E\left(x_{n:1}x_{n:1}^{⊤}\right)$, where *U* is unitary. Thus, $\sqrt{E\;\left(x_{n:1}x_{n:1}^{⊤}\right)}=U{\mathrm{diag}}_{1\leq j\leq nN}\;\;\left(\sqrt{\lambda_{j}\left(E\left(x_{n:1}x_{n:1}^{⊤}\right)\right)}\right)U^{*}$ and ${\left(\sqrt{E\;\left(x_{n:1}x_{n:1}^{⊤}\right)}\right)}^{-1}=U{\mathrm{diag}}_{1\leq j\leq nN}\;\;\left(\frac{1}{\sqrt{\lambda_{j}\left(E\left(x_{n:1}x_{n:1}^{⊤}\right)\right)}}\right)U^{*}$.

Since ${\left(\sqrt{E\;\left(x_{n:1}x_{n:1}^{⊤}\right)}\right)}^{-1}$ is Hermitian and $C_{E\left(x_{n:1}x_{n:1}^{⊤}\right)}$ is a positive definite matrix, ${\left(\sqrt{E\;\left(x_{n:1}x_{n:1}^{⊤}\right)}\right)}^{-1}C_{E\left(x_{n:1}x_{n:1}^{⊤}\right)}{\left(\sqrt{E\;\left(x_{n:1}x_{n:1}^{⊤}\right)}\right)}^{-1}$ is also a positive definite matrix.

From Equation (5), we have

(12) $$R_{x_{n:1}}\left(D\right)=\frac{1}{2nN}\ln\frac{\det\left(E\left(x_{n:1}x_{n:1}^{⊤}\right)\right)}{D^{nN}},$$

and applying the arithmetic mean-geometric mean inequality yields

$$\begin{array}{cc} 0 & \leq \frac{1}{2nN}\ln\frac{\det\left(C_{E\left(x_{n:1}x_{n:1}^{⊤}\right)}\right)}{D^{nN}}-R_{x_{n:1}}\left(D\right)\\ \; & =\frac{1}{2nN}\ln\frac{\det\left(C_{E\left(x_{n:1}x_{n:1}^{⊤}\right)}\right)}{\det\left(E(x_{n:1}x_{n:1}^{⊤})\right)}=\frac{1}{2nN}\ln\frac{\det\left(C_{E\left(x_{n:1}x_{n:1}^{⊤}\right)}\right)}{\det\left(\sqrt{E\;\left(x_{n:1}x_{n:1}^{⊤}\right)}\right)\det\left(\sqrt{E\;\left(x_{n:1}x_{n:1}^{⊤}\right)}\right)}\\ \; & =\frac{1}{2nN}\ln\left(\det\left({\left(\sqrt{E\;\left(x_{n:1}x_{n:1}^{⊤}\right)}\right)}^{-1}\right)\det\left(C_{E\left(x_{n:1}x_{n:1}^{⊤}\right)}\right)\det\left({\left(\sqrt{E\;\left(x_{n:1}x_{n:1}^{⊤}\right)}\right)}^{-1}\right)\right)\\ \; & =\frac{1}{2nN}\ln\det\left({\left(\sqrt{E\;\left(x_{n:1}x_{n:1}^{⊤}\right)}\right)}^{-1}C_{E\left(x_{n:1}x_{n:1}^{⊤}\right)}{\left(\sqrt{E\;\left(x_{n:1}x_{n:1}^{⊤}\right)}\right)}^{-1}\right)\\ \; & =\frac{1}{2nN}\ln\prod_{j=1}^{nN}\lambda_{j}\left({\left(\sqrt{E\;\left(x_{n:1}x_{n:1}^{⊤}\right)}\right)}^{-1}C_{E\left(x_{n:1}x_{n:1}^{⊤}\right)}{\left(\sqrt{E\;\left(x_{n:1}x_{n:1}^{⊤}\right)}\right)}^{-1}\right)\\ \; & \leq \frac{1}{2nN}\ln\left({\left(\frac{1}{nN}\sum_{j=1}^{nN}\lambda_{j}\left({\left(\sqrt{E\;\left(x_{n:1}x_{n:1}^{⊤}\right)}\right)}^{-1}C_{E\left(x_{n:1}x_{n:1}^{⊤}\right)}{\left(\sqrt{E\;\left(x_{n:1}x_{n:1}^{⊤}\right)}\right)}^{-1}\right)\right)}^{nN}\right)\\ \; & =\frac{1}{2}\ln\left(\frac{1}{nN}\mathrm{tr}\left({\left(\sqrt{E\;\left(x_{n:1}x_{n:1}^{⊤}\right)}\right)}^{-1}C_{E\left(x_{n:1}x_{n:1}^{⊤}\right)}{\left(\sqrt{E\;\left(x_{n:1}x_{n:1}^{⊤}\right)}\right)}^{-1}\right)\right)\\ \; & =\frac{1}{2}\ln\left(\frac{1}{nN}\mathrm{tr}\left(C_{E\left(x_{n:1}x_{n:1}^{⊤}\right)}{\left(\sqrt{E\;\left(x_{n:1}x_{n:1}^{⊤}\right)}\right)}^{-1}{\left(\sqrt{E\;\left(x_{n:1}x_{n:1}^{⊤}\right)}\right)}^{-1}\right)\right)\\ \; & =\frac{1}{2}\ln\left(\frac{1}{nN}\mathrm{tr}\left(C_{E\left(x_{n:1}x_{n:1}^{⊤}\right)}{\left(E\;\left(x_{n:1}x_{n:1}^{⊤}\right)\right)}^{-1}\right)\right)\\ \; & \leq \frac{1}{2}\ln\left(\frac{\sqrt{nN}}{nN}{∥C_{E\left(x_{n:1}x_{n:1}^{⊤}\right)}{\left(E\;\left(x_{n:1}x_{n:1}^{⊤}\right)\right)}^{-1}∥}_{F}\right)\\ \; & =\frac{1}{2}\ln\left(\frac{1}{\sqrt{nN}}{∥\left(C_{E\left(x_{n:1}x_{n:1}^{⊤}\right)}-E\;\left(x_{n:1}x_{n:1}^{⊤}\right)\right){\left(E\;\left(x_{n:1}x_{n:1}^{⊤}\right)\right)}^{-1}+I_{nN}∥}_{F}\right)\\ \; & \leq \frac{1}{2}\ln\left(\frac{1}{\sqrt{nN}}\left({∥\left(C_{E\left(x_{n:1}x_{n:1}^{⊤}\right)}-E\;\left(x_{n:1}x_{n:1}^{⊤}\right)\right){\left(E\;\left(x_{n:1}x_{n:1}^{⊤}\right)\right)}^{-1}∥}_{F}+\sqrt{nN}\right)\right)\\ \; & \leq \frac{1}{2}\ln\left(\frac{1}{\sqrt{nN}}\left({∥C_{E\left(x_{n:1}x_{n:1}^{⊤}\right)}-E\;\left(x_{n:1}x_{n:1}^{⊤}\right)∥}_{F}{∥{\left(E\;\left(x_{n:1}x_{n:1}^{⊤}\right)\right)}^{-1}∥}_{2}+\sqrt{nN}\right)\right)\\ \; & =\frac{1}{2}\ln\left(1+\frac{{∥E\;\left(x_{n:1}x_{n:1}^{⊤}\right)-C_{E\left(x_{n:1}x_{n:1}^{⊤}\right)}∥}_{F}}{\sqrt{nN}\lambda_{nN}\left(E\;\left(x_{n:1}x_{n:1}^{⊤}\right)\right)}\right). \end{array}$$

 □

In Equation (12), $R_{x_{n:1}}\left(D\right)$ is written in terms of $E\left(x_{n:1}x_{n:1}^{⊤}\right)$. ${\tilde{R}}_{x_{n:1}}\left(D\right)$ can be written in terms of $E\left(x_{n:1}x_{n:1}^{⊤}\right)$ and $V_{n}$ by using Lemma 2 and Equations (9) and (10).

As our coding strategy requires the computation of the block DFT, its computational complexity is O(nNlogn) whenever the FFT algorithm is used. We recall that the computational complexity of the optimal coding strategy for $x_{n:1}$ is $O(n^{2}N^{2})$ since it requires the computation of $U_{n}^{⊤}x_{n:1}$, where $U_{n}$ is a real orthogonal eigenvector matrix of $E\left(x_{n:1}x_{n:1}^{⊤}\right)$. Observe that such eigenvector matrix $U_{n}$ also needs to be computed, which further increases the complexity. Hence, the main advantage of our coding strategy is that it notably reduces the computational complexity of coding $x_{n:1}$. Moreover, our coding strategy does not require the knowledge of $E\left(x_{n:1}x_{n:1}^{⊤}\right)$. It only requires the knowledge of $E\left(\hat{y_{k}}{\hat{y_{k}}}^{⊤}\right)$, with $k\in \{\left\lceil \frac{n}{2}\right\rceil \ldots ,n\}$.

It should be mentioned that Equation (7) provides two upper bounds for the RDF of finite-length data blocks of a real zero-mean Gaussian *N*-dimensional vector source $\left\{x_{k}\right\}$. The greatest upper bound in Equation (7) was given in [11] for the case in which the random vector source $\left\{x_{k}\right\}$ is WSS, and therefore, the correlation matrix of the Gaussian vector, $E\left(x_{n:1}x_{n:1}^{⊤}\right)$, is a block Toeplitz matrix. Such upper bound was first presented by Pearl in [12] for the case in which the source is WSS and N=1. However, neither [11] nor [12] propose a coding strategy for $\left\{x_{k}\right\}$.

## 4. Optimality of the Proposed Coding Strategy for Gaussian AWSS Vector Sources

In this section (see Theorem 4), we show that our coding strategy is asymptotically optimal, i.e., we show that for large enough data blocks of a Gaussian AWSS vector source $\left\{x_{k}\right\}$, the rate of our coding strategy, presented in Section 3, tends to the RDF of the source.

We begin by introducing some notation. If $X:R\to C^{N\times N}$ is a continuous and 2π-periodic matrix-valued function of a real variable, we denote by $T_{n}\left(X\right)$ the n×n block Toeplitz matrix with N×N blocks given by

$$T_{n}\left(X\right)={\left(X_{j-k}\right)}_{j,k=1}^{n},$$

where ${\left\{X_{k}\right\}}_{k\in Z}$ is the sequence of Fourier coefficients of *X*:

$$X_{k}=\frac{1}{2\pi }\int_{0}^{2\pi }e^{-k\omega i}X\left(\omega \right)d\omega \;\forall k\in Z.$$

If $A_{n}$ and $B_{n}$ are nN×nN matrices for all n∈N, we write $\left\{A_{n}\right\}∼\left\{B_{n}\right\}$ when the sequences $\left\{A_{n}\right\}$ and $\left\{B_{n}\right\}$ are asymptotically equivalent (see ([13] (p. 5673))), that is, $\left\{∥A_{n}{∥}_{2}\right\}$ and $\left\{∥B_{n}{∥}_{2}\right\}$ are bounded and

$$\lim_{n\to \infty }\frac{∥A_{n}-B_{n}{∥}_{F}}{\sqrt{n}}=0.$$

The original definition of asymptotically equivalent sequences of matrices was given by Gray (see ([2] (Section 2.3)) or [4]) for N=1.

We now review the definition of the AWSS vector process given in ([1] (Definition 7.1)). This definition was first introduced for the scalar case N = 1 (see ([2] (Section 6)) or [3]).

**Definition** **1.** Let $X:R\to C^{N\times N}$, and suppose that it is continuous and 2π-periodic. A random N-dimensional vector process $\left\{x_{k}\right\}$ is said to be AWSS with asymptotic power spectral density (APSD) X if it has constant mean (i.e., $E\left(x_{k_{1}}\right)=E\left(x_{k_{2}}\right)$ for all $k_{1},k_{2}\in N)$ and $\left\{E\left(x_{n:1}x_{n:1}^{*}\right)\right\}∼\left\{T_{n}\left(X\right)\right\}$.

We recall that the RDF of $\left\{x_{k}\right\}$ is defined as $\lim_{n\to \infty }R_{x_{n:1}}\left(D\right)$.

**Theorem** **4.** *Let $\left\{x_{k}\right\}$ be a real zero-mean Gaussian AWSS N-dimensional vector process with APSD X. Suppose that $\inf_{n\in N}\lambda_{nN}\left(E\left(x_{n:1}x_{n:1}^{⊤}\right)\right)>0$. If $D\in \left(0,\inf_{n\in N}\lambda_{nN}\left(E\left(x_{n:1}x_{n:1}^{⊤}\right)\right)\right]$, then*

(13) $$\lim_{n\to \infty }R_{x_{n:1}}\left(D\right)=\lim_{n\to \infty }{\tilde{R}}_{x_{n:1}}\left(D\right)=\frac{1}{4\pi N}\int_{0}^{2\pi }\ln\frac{\det(X(\omega ))}{D^{N}}d\omega .$$

**Proof.**  We divide the proof into two steps:

*Step 1:* We show that $\lim_{n\to \infty }R_{x_{n:1}}\left(D\right)=\frac{1}{4\pi N}\int_{0}^{2\pi }\ln\frac{\det(X(\omega ))}{D^{N}}d\omega$. From Equation (12), ([1] (Theorem 6.6)), and ([14] (Proposition 2)) yields

$$\begin{array}{cc} \lim_{n\to \infty }R_{x_{n:1}}\left(D\right) & =\lim_{n\to \infty }\frac{1}{2nN}\ln\frac{\prod_{k=1}^{nN}\lambda_{k}\left(E\left(x_{n:1}x_{n:1}^{⊤}\right)\right)}{D^{nN}}=\lim_{n\to \infty }\frac{1}{2nN}\sum_{k=1}^{nN}\ln\frac{\lambda_{k}\left(E\left(x_{n:1}x_{n:1}^{⊤}\right)\right)}{D}\\ \; & =\frac{1}{4\pi }\int_{0}^{2\pi }\frac{1}{N}\sum_{k=1}^{N}\ln\frac{\lambda_{k}\left(X\left(\omega \right)\right)}{D}d\omega =\frac{1}{4\pi N}\int_{0}^{2\pi }\ln\frac{\det(X(\omega ))}{D^{N}}d\omega . \end{array}$$

*Step 2:* We prove that $\lim_{n\to \infty }R_{x_{n:1}}\left(D\right)=\lim_{n\to \infty }{\tilde{R}}_{x_{n:1}}\left(D\right)$. Applying Equations (7) and (8), we obtain

(14) $$\begin{array}{cc} 0\; & \leq \;{\tilde{R}}_{x_{n:1}}\left(D\right)-R_{x_{n:1}}\left(D\right)\;\leq \;\frac{1}{2nN}\ln\frac{\det\left(C_{E\left(x_{n:1}x_{n:1}^{⊤}\right)}\right)}{D^{nN}}-R_{x_{n:1}}\left(D\right)\\ \; & \leq \frac{1}{2}\ln\left(1+\frac{{∥E\left(x_{n:1}x_{n:1}^{⊤}\right)-C_{E\left(x_{n:1}x_{n:1}^{⊤}\right)}∥}_{F}}{\sqrt{nN}\lambda_{nN}\left(E\left(x_{n:1}x_{n:1}^{⊤}\right)\right)}\right)\\ \; & \leq \frac{1}{2}\ln\left(1+\frac{{∥E\left(x_{n:1}x_{n:1}^{⊤}\right)-C_{E\left(x_{n:1}x_{n:1}^{⊤}\right)}∥}_{F}}{\sqrt{nN}\inf_{m\in N}\lambda_{mN}\left(E\left(x_{m:1}x_{m:1}^{⊤}\right)\right)}\right)\;\forall n\in N. \end{array}$$

To finish the proof, we only need to show that

(15) $$\lim_{n\to \infty }\frac{{∥E\;\left(x_{n:1}x_{n:1}^{⊤}\right)-C_{E\left(x_{n:1}x_{n:1}^{⊤}\right)}∥}_{F}}{\sqrt{n}}=0.$$

Let $C_{n}\left(X\right)$ be the n×n block circulant matrix with N×N blocks defined in ([13] (p. 5674)), i.e.,

$$C_{n}\left(X\right)=\left(V_{n}⊗I_{N}\right){\mathrm{diag}}_{1\leq k\leq n}\left(X\left(\frac{2\pi (k-1)}{n}\right)\right){\left(V_{n}⊗I_{N}\right)}^{*}\;\forall n\in N.$$

Observe that

$$\begin{array}{cc} C_{C_{n}\left(X\right)}= & \left(V_{n}⊗I_{N}\right){\mathrm{diag}}_{1\leq k\leq n}\left({\left[{\left(V_{n}⊗I_{N}\right)}^{*}C_{n}\left(X\right)\left(V_{n}⊗I_{N}\right)\right]}_{k,k}\right){\left(V_{n}⊗I_{N}\right)}^{*}\\ = & \left(V_{n}⊗I_{N}\right){\mathrm{diag}}_{1\leq k\leq n}\left({\left[{\mathrm{diag}}_{1\leq j\leq n}\left(X\left(\frac{2\pi (j-1)}{n}\right)\right)\right]}_{k,k}\right){\left(V_{n}⊗I_{N}\right)}^{*}\\ = & \left(V_{n}⊗I_{N}\right){\mathrm{diag}}_{1\leq k\leq n}\left(X\left(\frac{2\pi (k-1)}{n}\right)\right){\left(V_{n}⊗I_{N}\right)}^{*}=C_{n}\left(X\right)\;\forall n\in N. \end{array}$$

Consequently, as the Frobenius norm is unitarily invariant, we have

$$\begin{array}{cc} \; & {∥C_{n}\left(X\right)-C_{E\left(x_{n:1}x_{n:1}^{⊤}\right)}∥}_{F}={∥C_{C_{n}\left(X\right)}-C_{E\left(x_{n:1}x_{n:1}^{⊤}\right)}∥}_{F}\\ \; & \;\;\;\;\;\;={∥\left(V_{n}⊗I_{N}\right){\mathrm{diag}}_{1\leq k\leq n}\left({\left[{\left(V_{n}⊗I_{N}\right)}^{*}\left(C_{n}\left(X\right)-E\left(x_{n:1}x_{n:1}^{⊤}\right)\right)\left(V_{n}⊗I_{N}\right)\right]}_{k,k}\right){\left(V_{n}⊗I_{N}\right)}^{*}∥}_{F}\\ \; & \;\;\;\;\;\;={∥{\mathrm{diag}}_{1\leq k\leq n}\left({\left[{\left(V_{n}⊗I_{N}\right)}^{*}\left(C_{n}\left(X\right)-E\left(x_{n:1}x_{n:1}^{⊤}\right)\right)\left(V_{n}⊗I_{N}\right)\right]}_{k,k}\right)∥}_{F}\\ \; & \;\;\;\;\;\;\leq {∥{\left(V_{n}⊗I_{N}\right)}^{*}\left(C_{n}\left(X\right)-E\left(x_{n:1}x_{n:1}^{⊤}\right)\right)\left(V_{n}⊗I_{N}\right)∥}_{F}={∥C_{n}\left(X\right)-E\left(x_{n:1}x_{n:1}^{⊤}\right)∥}_{F}\;\forall n\in N. \end{array}$$

Therefore,

(16) $$\begin{array}{cc} 0\leq & \frac{{∥E\left(x_{n:1}x_{n:1}^{⊤}\right)-C_{E\left(x_{n:1}x_{n:1}^{⊤}\right)}∥}_{F}}{\sqrt{n}}\leq \frac{{∥E\left(x_{n:1}x_{n:1}^{⊤}\right)-C_{n}\left(X\right)∥}_{F}}{\sqrt{n}}+\frac{{∥C_{n}\left(X\right)-C_{E\left(x_{n:1}x_{n:1}^{⊤}\right)}∥}_{F}}{\sqrt{n}}\\ \leq & 2\frac{{∥E\;\left(x_{n:1}x_{n:1}^{⊤}\right)-C_{n}\left(X\right)∥}_{F}}{\sqrt{n}}\leq 2\left(\frac{{∥E\;\left(x_{n:1}x_{n:1}^{⊤}\right)-T_{n}\left(X\right)∥}_{F}}{\sqrt{n}}+\frac{{∥T_{n}\left(X\right)-C_{n}\left(X\right)∥}_{F}}{\sqrt{n}}\right)\;\forall n\in N. \end{array}$$

Since $\left\{E\left(x_{n:1}x_{n:1}^{⊤}\right)\right\}∼\left\{T_{n}\left(X\right)\right\}$, Equation (16) and ([1] (Lemma 6.1)) yields Equation (15). □

Observe that the integral formula in Equation (13) provides the value of the RDF of the Gaussian AWSS vector source whenever $D\in \left(0,\inf_{n\in N}\lambda_{nN}\left(E\left(x_{n:1}x_{n:1}^{⊤}\right)\right)\right]$. An integral formula of such an RDF for any D>0 can be found in ([15] (Theorem 1)). It should be mentioned that ([15] (Theorem 1)) generalized the integral formulas previously given in the literature for the RDF of certain Gaussian AWSS sources, namely, WSS scalar sources [9], AR AWSS scalar sources [16], and AR AWSS vector sources of finite order [17].

## 5. Relevant AWSS Vector Sources

WSS, MA, AR, and ARMA vector processes are frequently used to model multivariate time series (see, e.g., [18]) that arise in any domain that involves temporal measurements. In this section, we show that our coding strategy is appropriate to encode the aforementioned vector sources whenever they are Gaussian and AWSS.

It should be mentioned that Gaussian AWSS MA vector (VMA) processes, Gaussian AWSS AR vector (VAR) processes, and Gaussian AWSS ARMA vector (VARMA) processes are frequently called Gaussian stationary VMA processes, Gaussian stationary VAR processes, and Gaussian stationary VARMA processes, respectively (see, e.g., [18]). However, they are asymptotically stationary but not stationary, because their corresponding correlation matrices are not block Toeplitz.

### 5.1. WSS Vector Sources

In this subsection (see Theorem 5), we give conditions under which our coding strategy is asymptotically optimal for WSS vector sources.

We first recall the well-known concept of WSS vector process.

**Definition** **2.** Let $X:R\to C^{N\times N}$, and suppose that it is continuous and 2π-periodic. A random N-dimensional vector process $\left\{x_{k}\right\}$ is said to be WSS (or weakly stationary) with PSD X if it has constant mean and $\left\{E\left(x_{n:1}x_{n:1}^{*}\right)\right\}=\left\{T_{n}\left(X\right)\right\}$.

**Theorem** **5.** *Let $\left\{x_{k}\right\}$ be a real zero-mean Gaussian WSS N-dimensional vector process with PSD X. Suppose that $\min_{\omega \in [0,2\pi ]}\lambda_{N}\left(X(\omega )\right)>0$ (or equivalently, det(X(ω))≠0 for all ω∈R). If $D\in \left(0,\min_{\omega \in [0,2\pi ]}\lambda_{N}\left(X(\omega )\right)\right]$, then*

$$\lim_{n\to \infty }R_{x_{n:1}}\left(D\right)=\lim_{n\to \infty }{\tilde{R}}_{x_{n:1}}\left(D\right)=\frac{1}{4\pi N}\int_{0}^{2\pi }\ln\frac{\det\left(X(\omega )\right)}{D^{N}}d\omega .$$

**Proof.**  Applying ([1] (Lemma 3.3)) and ([1] (Theorem 4.3)) yields $\left\{E\left(x_{n:1}x_{n:1}^{⊤}\right)\right\}=\left\{T_{n}\left(X\right)\right\}∼\left\{T_{n}\left(X\right)\right\}$. Theorem 5 now follows from ([14] (Proposition 3)) and Theorem 4. □

Theorem 5 was presented in [5] for the case N=1 (i.e., just for WSS sources but not for vector WSS sources).

### 5.2. VMA Sources

In this subsection (see Theorem 6), we give conditions under which our coding strategy is asymptotically optimal for VMA sources.

We start by reviewing the concept of VMA process.

**Definition** **3.** *A real zero-mean random N-dimensional vector process $\left\{x_{k}\right\}$ is said to be MA if*

$$x_{k}=w_{k}+\sum_{j=1}^{k-1}G_{-j}w_{k-j}\;\forall k\in N,$$

*where $G_{-j}$, j∈N, are real N×N matrices, $\left\{w_{k}\right\}$ is a real zero-mean random N-dimensional vector process, and $E\left(w_{k_{1}}w_{k_{2}}^{⊤}\right)=\delta_{k_{1},k_{2}}\Lambda$ for all $k_{1},k_{2}\in N$ with Λ being a real N×N positive definite matrix. If there exists q∈N such that $G_{-j}=0_{N\times N}$ for all j>q, then $\left\{x_{k}\right\}$ is called a VMA(q) process.*

**Theorem** **6.** *Let $\left\{x_{k}\right\}$ be as in Definition 3. Assume that ${\left\{G_{k}\right\}}_{k=-\infty }^{\infty }$, with $G_{0}=I_{N}$ and $G_{k}=0_{N\times N}$ for all k∈N, is the sequence of Fourier coefficients of a function $G:R\to C^{N\times N}$, which is continuous and 2π-periodic. Suppose that $\left\{T_{n}\left(G\right)\right\}$ is stable (that is, $\left\{∥{\left(T_{n}\left(G\right)\right)}^{-1}{∥}_{2}\right\}$ is bounded). If $\left\{x_{k}\right\}$ is Gaussian and $D\in \left(0,\inf_{n\in N}\lambda_{nN}\left(E\left(x_{n:1}x_{n:1}^{⊤}\right)\right)\right]$, then*

(17) $$\lim_{n\to \infty }R_{x_{n:1}}\left(D\right)=\lim_{n\to \infty }{\tilde{R}}_{x_{n:1}}\left(D\right)=\frac{1}{2N}\ln\frac{\det(\Lambda )}{D^{N}}.$$

*Moreover, $R_{x_{n:1}}\left(D\right)=\frac{1}{2N}\ln\frac{\det(\Lambda )}{D^{N}}$ for all n∈N.*

**Proof.**  We divide the proof into three steps:

*Step 1:* We show that $\det\;\left(\;E\;\left(x_{n:1}x_{n:1}^{⊤}\right)\;\right)={\left(\;\det(\;\Lambda \;)\;\right)}^{n}$ for all n∈N. From ([15] (Equation (A3))) we have that $\left\{E\left(x_{n:1}x_{n:1}^{⊤}\right)\right\}=\left\{T_{n}\left(G\right)T_{n}\left(\Lambda \right){\left(T_{n}\left(G\right)\right)}^{*}\right\}$. Consequently,

$$\begin{array}{cc} \det\;\left(\;E\;\left(x_{n:1}x_{n:1}^{⊤}\right)\;\right) & \;=\;\det\left(\;T_{n}\left(G\right)\;\right)\det\left(\;T_{n}\left(\Lambda \right)\;\right)\bar{\det\left(\;T_{n}\left(G\right)\;\right)}\;=\;{\left|\;\det\left(\;T_{n}\left(G\right)\;\right)\;\right|}^{2}{\left(\;\det(\;\Lambda \;)\;\right)}^{n}\;={\left(\;\det(\;\Lambda \;)\;\right)}^{n}\;\forall n\in N. \end{array}$$

*Step 2:* We prove the first equality in Equation (17). Applying ([15] (Theorem 2)), we obtain that $\left\{x_{k}\right\}$ is AWSS. From Theorem 4, we only need to show that $\inf_{n\in N}\lambda_{nN}\left(E\left(x_{n:1}x_{n:1}^{⊤}\right)\right)>0$. We have

$$\begin{array}{cc} \; & \lambda_{nN}\left(E\left(x_{n:1}x_{n:1}^{⊤}\right)\right)=\frac{1}{\lambda_{1}\left({\left(E\left(x_{n:1}x_{n:1}^{⊤}\right)\right)}^{-1}\right)}=\frac{1}{{∥{\left(E\left(x_{n:1}x_{n:1}^{⊤}\right)\right)}^{-1}∥}_{2}}=\frac{1}{{∥{\left(T_{n}\left(G\right)T_{n}\left(\Lambda \right){\left(T_{n}\left(G\right)\right)}^{*}\right)}^{-1}∥}_{2}}\\ \; & \;\;\;\;\;\;\;\;\;\;\;=\frac{1}{{∥{\left({\left(T_{n}\left(G\right)\right)}^{-1}\right)}^{*}T_{n}\left(\Lambda^{-1}\right){\left(T_{n}\left(G\right)\right)}^{-1}∥}_{2}}\geq \frac{1}{{∥{\left({\left(T_{n}\left(G\right)\right)}^{-1}\right)}^{*}∥}_{2}{∥T_{n}\left(\Lambda^{-1}\right)∥}_{2}{∥{\left(T_{n}\left(G\right)\right)}^{-1}∥}_{2}}\\ \; & \;\;\;\;\;\;\;\;\;\;\;=\frac{1}{{∥{\left(T_{n}\left(G\right)\right)}^{-1}∥}_{2}^{2}\lambda_{1}\left(\Lambda^{-1}\right)}=\frac{\lambda_{N}\left(\Lambda \right)}{{∥{\left(T_{n}\left(G\right)\right)}^{-1}∥}_{2}^{2}}\geq \frac{\lambda_{N}\left(\Lambda \right)}{{\left(\sup_{m\in N}{∥{\left(T_{m}\left(G\right)\right)}^{-1}∥}_{2}\right)}^{2}}>0\;\forall n\in N. \end{array}$$

*Step 3:* We show that $R_{x_{n:1}}\left(D\right)=\frac{1}{2N}\ln\frac{\det(\Lambda )}{D^{N}}$ for all n∈N. Applying Equation (12) yields

$$\begin{array}{c} R_{x_{n:1}}\left(D\right)=\frac{1}{2nN}\ln\frac{{\left(\det(\Lambda )\right)}^{n}}{D^{nN}}=\frac{1}{2N}\ln\frac{\det(\Lambda )}{D^{N}}\;\forall n\in N. \end{array}$$

 □

### 5.3. VAR AWSS Sources

In this subsection (see Theorem 7), we give conditions under which our coding strategy is asymptotically optimal for VAR sources.

We first recall the concept of VAR process.

**Definition** **4.** *A real zero-mean random N-dimensional vector process $\left\{x_{k}\right\}$ is said to be AR if*

$$x_{k}=w_{k}-\sum_{j=1}^{k-1}F_{-j}x_{k-j}\;\forall k\in N,$$

*where $F_{-j}$, j∈N, are real N×N matrices, $\left\{w_{k}\right\}$ is a real zero-mean random N-dimensional vector process, and $E\left(w_{k_{1}}w_{k_{2}}^{⊤}\right)=\delta_{k_{1},k_{2}}\Lambda$ for all $k_{1},k_{2}\in N$ with Λ being a real N×N positive definite matrix. If there exists p∈N such that $F_{-j}=0_{N\times N}$ for all j>p, then $\left\{x_{k}\right\}$ is called a VAR(p) process.*

**Theorem** **7.** *Let $\left\{x_{k}\right\}$ be as in Definition 4. Assume that ${\left\{F_{k}\right\}}_{k=-\infty }^{\infty }$, with $F_{0}=I_{N}$ and $F_{k}=0_{N\times N}$ for all k∈N, is the sequence of Fourier coefficients of a function $F:R\to C^{N\times N}$, which is continuous and 2π-periodic. Suppose that $\left\{T_{n}\left(F\right)\right\}$ is stable and $\det\left(F(\omega )\right)\neq 0$ for all ω∈R. If $\left\{x_{k}\right\}$ is Gaussian and $D\in \left(0,\inf_{n\in N}\lambda_{nN}\left(E\left(x_{n:1}x_{n:1}^{⊤}\right)\right)\right]$, then*

(18) $$\lim_{n\to \infty }R_{x_{n:1}}\left(D\right)=\lim_{n\to \infty }{\tilde{R}}_{x_{n:1}}\left(D\right)=\frac{1}{2N}\ln\frac{\det(\Lambda )}{D^{N}}.$$

*Moreover, $R_{x_{n:1}}\left(D\right)=\frac{1}{2N}\ln\frac{\det(\Lambda )}{D^{N}}$ for all n∈N.*

**Proof.**  We divide the proof into three steps:

*Step 1:* We show that $\det\;\left(\;E\;\left(x_{n:1}x_{n:1}^{⊤}\right)\;\right)={\left(\;\det(\;\Lambda \;)\;\right)}^{n}$ for all n∈N. From ([19] (Equation (19))), we have that $\left\{E\left(x_{n:1}x_{n:1}^{⊤}\right)\right\}=\left\{{\left(T_{n}\left(F\right)\right)}^{-1}T_{n}\left(\Lambda \right){\left({\left(T_{n}\left(F\right)\right)}^{*}\right)}^{-1}\right\}$. Consequently,

$$\begin{array}{cc} \det\;\left(\;E\;\left(x_{n:1}x_{n:1}^{⊤}\right)\;\right) & \;=\;\frac{\det\left(T_{n}\left(\Lambda \right)\right)}{\det\left(T_{n}\left(F\right)\right)\det\left({\left(T_{n}\left(F\right)\right)}^{*}\right)}\;=\;\frac{{\left(\det(\Lambda )\right)}^{n}}{|\det\left(T_{n}\left(F\right)\right){|}^{2}}\;=\;{\left(\det(\Lambda )\right)}^{n}\;\forall n\in N. \end{array}$$

*Step 2:* We prove the first equality in Equation (18). Applying ([15] (Theorem 3)), we obtain that $\left\{x_{k}\right\}$ is AWSS. From Theorem 4, we only need to show that $\inf_{n\in N}\lambda_{nN}\left(E\left(x_{n:1}x_{n:1}^{⊤}\right)\right)>0$. Applying ([1] (Theorem 4.3)) yields

$$\begin{array}{cc} \lambda_{nN}\left(E\left(x_{n:1}x_{n:1}^{⊤}\right)\right) & =\frac{1}{{∥{\left(E\left(x_{n:1}x_{n:1}^{⊤}\right)\right)}^{-1}∥}_{2}}=\frac{1}{{∥{\left({\left(T_{n}\left(F\right)\right)}^{-1}T_{n}\left(\Lambda \right){\left({\left(T_{n}\left(F\right)\right)}^{*}\right)}^{-1}\right)}^{-1}∥}_{2}}\\ \; & \geq \frac{\lambda_{N}\left(\Lambda \right)}{{∥T_{n}\left(F\right)∥}_{2}^{2}}\geq \frac{\lambda_{N}\left(\Lambda \right)}{{\left(\sup_{m\in N}{∥T_{m}\left(F\right)∥}_{2}\right)}^{2}}>0\;\forall n\in N. \end{array}$$

*Step 3:* We show that $R_{x_{n:1}}\left(D\right)=\frac{1}{2N}\ln\frac{\det(\Lambda )}{D^{N}}$ for all n∈N. This can be directly obtained from Equation (12). □

Theorem 7 was presented in [6] for the case of N=1 (i.e., just for AR sources but not for VAR sources).

### 5.4. VARMA AWSS Sources

In this subsection (see Theorem 8), we give conditions under which our coding strategy is asymptotically optimal for VARMA sources.

We start by reviewing the concept of VARMA process.

**Definition** **5.** *A real zero-mean random N-dimensional vector process $\left\{x_{k}\right\}$ is said to be ARMA if*

$$x_{k}=w_{k}+\sum_{j=1}^{k-1}G_{-j}w_{k-j}-\sum_{j=1}^{k-1}F_{-j}x_{k-j}\;\forall k\in N,$$

*where $G_{-j}$ and $F_{-j}$, j∈N, are real N×N matrices, $\left\{w_{k}\right\}$ is a real zero-mean random N-dimensional vector process, and $E\left(w_{k_{1}}w_{k_{2}}^{⊤}\right)=\delta_{k_{1},k_{2}}\Lambda$ for all $k_{1},k_{2}\in N$ with Λ being a real N×N positive definite matrix. If there exists p,q∈N such that $F_{-j}=0_{N\times N}$ for all j>p and $G_{-j}=0_{N\times N}$ for all j>q, then $\left\{x_{k}\right\}$ is called a VARMA(p,q) process (or a VARMA process of (finite) order (p,q)).*

**Theorem** **8.** *Let $\left\{x_{k}\right\}$ be as in Definition 5. Assume that ${\left\{G_{k}\right\}}_{k=-\infty }^{\infty }$, with $G_{0}=I_{N}$ and $G_{k}=0_{N\times N}$ for all k∈N, is the sequence of Fourier coefficients of a function $G:R\to C^{N\times N}$ which is continuous and 2π-periodic. Suppose that ${\left\{F_{k}\right\}}_{k=-\infty }^{\infty }$, with $F_{0}=I_{N}$ and $F_{k}=0_{N\times N}$ for all k∈N, is the sequence of Fourier coefficients of a function $F:R\to C^{N\times N}$ which is continuous and 2π-periodic. Assume that $\left\{T_{n}\left(G\right)\right\}$ and $\left\{T_{n}\left(F\right)\right\}$ are stable, and $\det\left(F(\omega )\right)\neq 0$ for all ω∈R. If $\left\{x_{k}\right\}$ is Gaussian and $D\in \left(0,\inf_{n\in N}\lambda_{nN}\left(E\left(x_{n:1}x_{n:1}^{⊤}\right)\right)\right]$, then*

(19) $$\lim_{n\to \infty }R_{x_{n:1}}\left(D\right)=\lim_{n\to \infty }{\tilde{R}}_{x_{n:1}}\left(D\right)=\frac{1}{2N}\ln\frac{\det(\Lambda )}{D^{N}}.$$

*Moreover, $R_{x_{n:1}}\left(D\right)=\frac{1}{2N}\ln\frac{\det(\Lambda )}{D^{N}}$ for all n∈N.*

**Proof.**  We divide the proof into three steps:

*Step 1:* We show that $\det\;\left(\;E\;\left(x_{n:1}x_{n:1}^{⊤}\right)\;\right)={\left(\;\det(\;\Lambda \;)\;\right)}^{n}$ for all n∈N. From ([15] (Appendix D)) and ([1] (Lemma 4.2)), we have that $\left\{E\left(x_{n:1}x_{n:1}^{⊤}\right)\right\}=\left\{{\left(T_{n}\left(F\right)\right)}^{-1}T_{n}\left(G\right)T_{n}\left(\Lambda \right){\left(T_{n}\left(G\right)\right)}^{*}{\left({\left(T_{n}\left(F\right)\right)}^{*}\right)}^{-1}\right\}$. Consequently,

$$\begin{array}{cc} \det\;\left(\;E\;\left(x_{n:1}x_{n:1}^{⊤}\right)\;\right) & \;=\;\frac{|\det\left(T_{n}\left(G\right)\right){|}^{2}{\left(\det(\Lambda )\right)}^{n}}{|\det\left(T_{n}\left(F\right)\right){|}^{2}}\;=\;{\left(\det(\Lambda )\right)}^{n}\;\forall n\in N. \end{array}$$

*Step 2:* We prove the first equality in Equation (19). Applying ([15] (Theorem 3)), we obtain that $\left\{x_{k}\right\}$ is AWSS. From Theorem 4, we only need to show that $\inf_{n\in N}\lambda_{nN}\left(E\left(x_{n:1}x_{n:1}^{⊤}\right)\right)>0$. Applying ([1] (Theorem 4.3)) yields

$$\begin{array}{cc} \; & \lambda_{nN}\left(E\left(x_{n:1}x_{n:1}^{⊤}\right)\right)=\frac{1}{{∥{\left(E\left(x_{n:1}x_{n:1}^{⊤}\right)\right)}^{-1}∥}_{2}}=\frac{1}{{∥{\left({\left(T_{n}\left(F\right)\right)}^{-1}T_{n}\left(G\right)T_{n}\left(\Lambda \right){\left(T_{n}\left(G\right)\right)}^{*}{\left({\left(T_{n}\left(F\right)\right)}^{*}\right)}^{-1}\right)}^{-1}∥}_{2}}\\ \; & \;\;\;\;\;\;\;\;\;\;\;\geq \frac{\lambda_{N}\left(\Lambda \right)}{{∥T_{n}\left(F\right)∥}_{2}^{2}{∥{\left(T_{n}\left(G\right)\right)}^{-1}∥}_{2}^{2}}\geq \frac{\lambda_{N}\left(\Lambda \right)}{{\left(\sup_{m\in N}{∥T_{m}\left(F\right)∥}_{2}\right)}^{2}{\left(\sup_{m\in N}{∥{\left(T_{m}\left(G\right)\right)}^{-1}∥}_{2}\right)}^{2}}>0\;\forall n\in N. \end{array}$$

*Step 3:* We show that $R_{x_{n:1}}\left(D\right)=\frac{1}{2N}\ln\frac{\det(\Lambda )}{D^{N}}$ for all n∈N. This can be directly obtained from Equation (12). □

## 6. Numerical Examples

We first consider four AWSS vector processes, namely, we consider the zero-mean WSS vector process in ([20] (Section 4)), the VMA(1) process in ([18] (Example 2.1)), the VAR(1) process in ([18] (Example 2.3)), and the VARMA(1,1) process in ([18] (Example 3.2)). In ([20] (Section 4)), N=2 and the Fourier coefficients of its PSD *X* are

$$X_{0}=\left(\begin{array}{cc} 2.0002 & 0.7058\\ 0.7058 & 2.0000 \end{array}\right),\;X_{-1}=X_{1}^{*}=\left(\begin{array}{cc} -0.3542 & 0.1016\\ 0.1839 & -0.2524 \end{array}\right),\;X_{-2}=X_{2}^{*}=\left(\begin{array}{cc} -0.0923 & 0.0153\\ 0.1490 & 0.0696 \end{array}\right),$$

$$X_{-3}=X_{3}^{*}=\left(\begin{array}{cc} -0.1443 & -0.0904\\ 0.0602 & 0.0704 \end{array}\right),\;X_{-4}=X_{4}^{*}=\left(\begin{array}{cc} -0.0516 & -0.0603\\ 0 & 0 \end{array}\right),$$

and $X_{j}=0_{2\times 2}$ with |j|>4. In ([18] (Example 2.1)), N=2, $G_{-1}$ is given by

(20) $$\left(\begin{array}{cc} -0.8 & -0.7\\ 0.4 & -0.6 \end{array}\right),$$

$G_{-j}=0_{2\times 2}$ for all j∈N, and

(21) $$\Lambda =\left(\begin{array}{cc} 4 & 1\\ 1 & 2 \end{array}\right).$$

In ([18] (Example 2.3)), N=2, $F_{-j}=0_{2\times 2}$ for all j∈N, and $F_{-1}$ and Λ are given by Equations (20) and (21), respectively. In ([18] (Example 3.2)), N=2,

$$G_{-1}=\left(\begin{array}{cc} 0.6 & -0.3\\ -0.3 & -0.6 \end{array}\right),\;F_{-1}=\left(\begin{array}{cc} -1.2 & 0.5\\ -0.6 & -0.3 \end{array}\right),\;\Lambda =\left(\begin{array}{cc} 1 & 0.5\\ 0.5 & 1.25 \end{array}\right),$$

$G_{-j}=0_{2\times 2}$ for all j∈N, and $F_{-j}=0_{2\times 2}$ for all j∈N.

**Figure 2:**
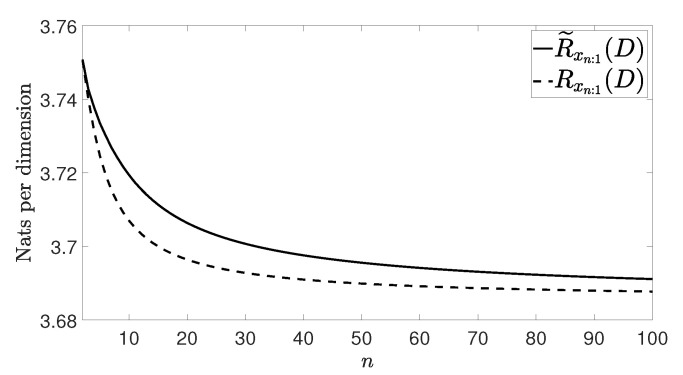
Considered rates for the wide sense stationary (WSS) vector process in ([20] (Section 4)).

**Figure 3:**
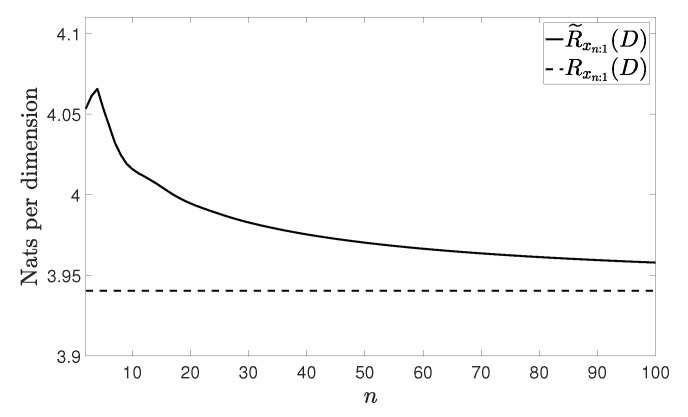
Considered rates for the VMA(1) process in ([18] (Example 2.1)).

**Figure 4:**
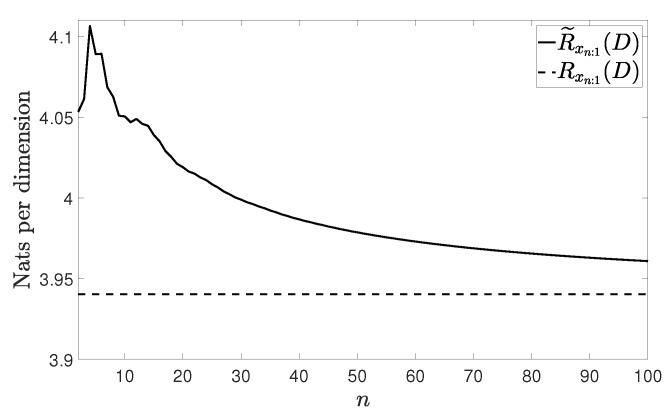
Considered rates for the VAR(1) process in ([18] (Example 2.3)).

**Figure 5:**
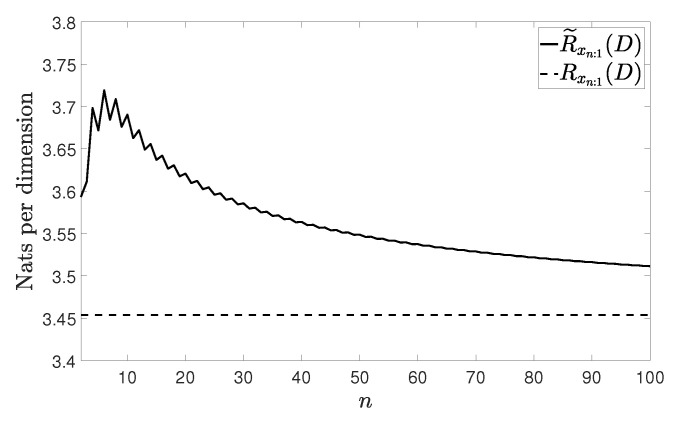
Considered rates for the VARMA(1,1) process in ([18] (Example 3.2)).

Figure 2, Figure 3, Figure 4 and Figure 5 show $R_{x_{n:1}}\left(D\right)$ and ${\tilde{R}}_{x_{n:1}}\left(D\right)$ with n≤100 and D=0.001 for the four vector processes considered, by assuming that they are Gaussian. The figures bear evidence of the fact that the rate of our coding strategy tends to the RDF of the source.

**Figure 6:**
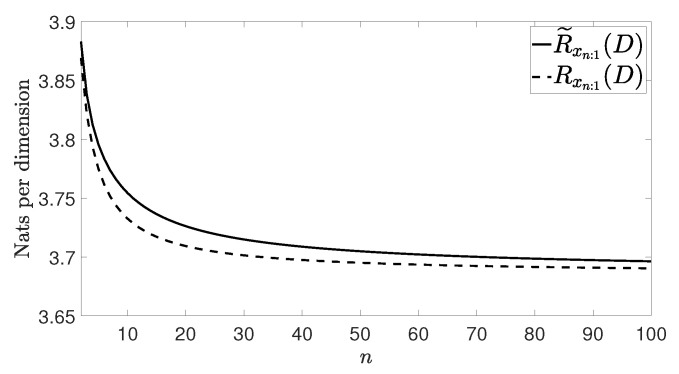
Considered rates for the perturbed WSS vector process with D = 0.001.

We finish with a numerical example to explore how our method performs in the presence of a perturbation. Specifically, we consider a perturbed version of the WSS vector process in ([20] (Section 4)) (Figure 6). The correlation matrices of the perturbed process are

$$T_{n}\left(X\right)+\left(\begin{array}{cc} 0_{2n-2\times 2n-2} & 0_{2n-2\times 2}\\ 0_{2\times 2n-2} & I_{2} \end{array}\right),\;n\in N.$$

## 7. Conclusions

The computational complexity of coding finite-length data blocks of Gaussian *N*-dimensional vector sources can be reduced by using the low-complexity coding strategy presented here instead of the optimal coding strategy. Specifically, the computational complexity is reduced from $O(n^{2}N^{2})$ to O(nNlogn), where *n* is the length of the data blocks. Moreover, our coding strategy is asymptotically optimal (i.e., the rate of our coding strategy tends to the RDF of the source) whenever the Gaussian vector source is AWSS and the considered data blocks are large enough. Besides being a low-complexity strategy, it does not require the knowledge of the correlation matrix of such data blocks. Furthermore, our coding strategy is appropriate to encode the most relevant Gaussian vector sources, namely, WSS, MA, AR, and ARMA vector sources.

## Appendix A. Proof of Lemma 1

**Proof.** (1) ⇒(2) We have

$$\begin{array}{ccc} y_{k}\;\;\; & = & \;\;\;{\left[y_{n:1}\right]}_{n-k+1,1}=\sum_{j=1}^{n}{\left[V_{n}^{*}\;⊗\;I_{N}\right]}_{n-k+1,j}{\left[x_{n:1}\right]}_{j,1}=\sum_{j=1}^{n}{\left[V_{n}^{*}\right]}_{n-k+1,j}I_{N}{\left[x_{n:1}\right]}_{j,1}=\sum_{j=1}^{n}\bar{{\left[V_{n}\right]}_{j,n-k+1}}{\left[x_{n:1}\right]}_{j,1}\\ \;\;\; & = & \;\;\;\frac{1}{\sqrt{n}}\sum_{j=1}^{n}e^{\frac{2\pi (j-1)(n-k)}{n}i}{\left[x_{n:1}\right]}_{j,1}=\frac{1}{\sqrt{n}}\sum_{j=1}^{n}e^{2\pi (j-1)i}e^{-\frac{2\pi (j-1)k}{n}i}{\left[x_{n:1}\right]}_{j,1}=\frac{1}{\sqrt{n}}\sum_{j=1}^{n}e^{-\frac{2\pi (j-1)k}{n}i}{\left[x_{n:1}\right]}_{j,1}\\ \;\;\; & = & \;\;\;\bar{\frac{1}{\sqrt{n}}\sum_{j=1}^{n}e^{\frac{2\pi (j-1)k}{n}i}{\left[x_{n:1}\right]}_{j,1}}=\bar{y_{n-k}} \end{array}$$

for all k∈{1,…,n−1} and $y_{n}=\frac{1}{\sqrt{n}}\sum_{j=1}^{n}{\left[x_{n:1}\right]}_{j,1}\in R^{N\times 1}$. (2)⇒(1) Since $V_{n}⊗I_{N}$ is a unitary matrix and

$$\begin{array}{c} {\left[V_{n}\right]}_{k,n-j+1}=\frac{1}{\sqrt{n}}e^{-\frac{2\pi (k-1)(n-j)}{n}i}=\frac{1}{\sqrt{n}}e^{-2\pi (k-1)i}e^{\frac{2\pi (k-1)j}{n}i}=\bar{{\left[V_{n}\right]}_{k,j+1}} \end{array}$$

for all k∈{1,…,n} and j∈{1,…,n−1}, we conclude that

xk=[xn:1]n−k+1,1=[Vn⊗INyn:1]n−k+1,1=∑j=1nVnn−k+1,jyn:1j,1=∑j=1nVnn−k+1,jyn−j+1=Vnn−k+1,1yn+∑h=1n−1Vnn−k+1,n−h+1yh=1nyn+∑h=1⌈n2⌉−1Vnn−k+1,n−h+1yh+Vnn−k+1,n−h+1¯yh¯+1+(−1)n2Vnn−k+1,⌈n2⌉+1y⌈n2⌉=1nyn+∑h=1⌈n2⌉−1Vnn−k+1,n−h+1yh+Vnn−k+1,n−h+1yh¯+1+(−1)n21ne−π(n−k)iy⌈n2⌉=1nyn+2∑h=1⌈n2⌉−1ReVnn−k+1,n−h+1yh+1+(−1)n2(−1)n−kny⌈n2⌉∈RN×1

for all k∈{1,…,n}. □

## Appendix B. Proof of Theorem 1

**Proof.** Fix k∈{1,…,n}. Let $E\left(x_{n:1}x_{n:1}^{*}\right)=U{\mathrm{diag}}_{1\leq j\leq nN}\;\;\left(\lambda_{j}\left(E\left(x_{n:1}x_{n:1}^{*}\right)\right)\right)U^{-1}$ and $E\left(x_{k}x_{k}^{*}\right)=W{\mathrm{diag}}_{1\leq j\leq N}\;\;\left(\lambda_{j}\left(E(x_{k}x_{k}^{*}\right)\right)W^{-1}$ be an eigenvalue decomposition (EVD) of $E\left(x_{n:1}x_{n:1}^{*}\right)$ and $E(x_{k}x_{k}^{*})$, respectively. We can assume that the eigenvector matrices *U* and *W* are unitary. We have

$$\begin{array}{ccc} \lambda_{j}\left(E(x_{k}x_{k}^{*})\right) & = & {\left[W^{*}E\left(x_{k}x_{k}^{*}\right)W\right]}_{j,j}=\sum_{h=1}^{N}{\left[W^{*}\right]}_{j,h}\sum_{l=1}^{N}{\left[E(x_{k}x_{k}^{*})\right]}_{h,l}{\left[W\right]}_{l,j}\\ & = & \sum_{h=1}^{N}{\left[W^{*}\right]}_{j,h}\sum_{l=1}^{N}{\left[E\left(x_{n:1}x_{n:1}^{*}\right)\right]}_{(n-k)N+h,(n-k)N+l}{\left[W\right]}_{l,j}\\ & = & \sum_{h=1}^{N}{\left[W^{*}\right]}_{j,h}\sum_{l=1}^{N}{\left[U{\mathrm{diag}}_{1\leq p\leq nN}\left(\lambda_{p}\left(E\left(x_{n:1}x_{n:1}^{*}\right)\right)\right)U^{*}\right]}_{(n-k)N+h,(n-k)N+l}{\left[W\right]}_{l,j}\\ & = & \sum_{h=1}^{N}{\left[W^{*}\right]}_{j,h}\sum_{l=1}^{N}\left(\sum_{p=1}^{nN}{\left[U\right]}_{(n-k)N+h,p}\lambda_{p}\left(E\left(x_{n:1}x_{n:1}^{*}\right)\right){\left[U^{*}\right]}_{p,(n-k)N+l}\right){\left[W\right]}_{l,j}\\ & = & \sum_{p=1}^{nN}\lambda_{p}\left(E\left(x_{n:1}x_{n:1}^{*}\right)\right)\sum_{h=1}^{N}\bar{{\left[W\right]}_{h,j}}{\left[U\right]}_{(n-k)N+h,p}\sum_{l=1}^{N}\bar{{\left[U\right]}_{(n-k)N+l,p}}{\left[W\right]}_{l,j}\\ & = & \sum_{p=1}^{nN}\lambda_{p}\left(E\left(x_{n:1}x_{n:1}^{*}\right)\right)\left(\sum_{h=1}^{N}\bar{{\left[W\right]}_{h,j}}{\left[U\right]}_{(n-k)N+h,p}\right)\bar{\left(\sum_{l=1}^{N}\bar{{\left[W\right]}_{l,j}}{\left[U\right]}_{(n-k)N+l,p}\right)}\\ & = & \sum_{p=1}^{nN}\lambda_{p}\left(E\left(x_{n:1}x_{n:1}^{*}\right)\right){\left|\sum_{h=1}^{N}\bar{{\left[W\right]}_{h,j}}{\left[U\right]}_{(n-k)N+h,p}\right|}^{2}, \end{array}$$

and consequently,

$$\begin{array}{cc} \lambda_{nN}\left(E\left(x_{n:1}x_{n:1}^{*}\right)\right)\sum_{p=1}^{nN}{\left|\sum_{h=1}^{N}\bar{{\left[W\right]}_{h,j}}{\left[U\right]}_{(n-k)N+h,p}\right|}^{2} & \leq \lambda_{j}\left(E(x_{k}x_{k}^{*})\right)\leq \\ \; & \lambda_{1}\left(E\left(x_{n:1}x_{n:1}^{*}\right)\right)\sum_{p=1}^{nN}{\left|\sum_{h=1}^{N}\bar{{\left[W\right]}_{h,j}}{\left[U\right]}_{(n-k)N+h,p}\right|}^{2} \end{array}$$

for all j∈{1,…,N}. Therefore, since

$$\begin{array}{cc} \sum_{p=1}^{nN} & {\left|\sum_{h=1}^{N}\bar{{\left[W\right]}_{h,j}}{\left[U\right]}_{(n-k)N+h,p}\right|}^{2}\;=\;\sum_{p=1}^{nN}\sum_{h=1}^{N}\bar{{\left[W\right]}_{h,j}}{\left[U\right]}_{(n-k)N+h,p}\sum_{l=1}^{N}\bar{{\left[U\right]}_{(n-k)N+l,p}}{\left[W\right]}_{l,j}\\ \; & \;=\;\sum_{h=1}^{N}{\left[W^{*}\right]}_{j,h}\;\sum_{l=1}^{N}\;\sum_{p=1}^{nN}{\left[U\right]}_{(n-k)N+h,p}{\left[U^{*}\right]}_{p,(n-k)N+l}{\left[W\right]}_{l,j}\;=\;\sum_{h=1}^{N}{\left[W^{*}\right]}_{j,h}\;\sum_{l=1}^{N}{\left[UU^{*}\right]}_{(n-k)N+h,(n-k)N+l}{\left[W\right]}_{l,j}\\ \; & \;=\;\sum_{h=1}^{N}{\left[W^{*}\right]}_{j,h}\sum_{l=1}^{N}{\left[I_{nN}\right]}_{(n-k)N+h,(n-k)N+l}{\left[W\right]}_{l,j}\;=\;\sum_{h=1}^{N}{\left[W^{*}\right]}_{j,h}{\left[W\right]}_{h,j}\;=\;{\left[W^{*}W\right]}_{j,j}\;=\;{\left[I_{N}\right]}_{j,j}=1, \end{array}$$

Equation (2) holds. We now prove Equation (3). Let $E\left(y_{k}y_{k}^{*}\right)=M{\mathrm{diag}}_{1\leq j\leq N}\;\;\left(\lambda_{j}\left(E(y_{k}y_{k}^{*}\right)\right)M^{-1}$ be an EVD of $E(y_{k}y_{k}^{*})$, where *M* is unitary. We have

$$\begin{array}{ccc} \lambda_{j}\left(E(y_{k}y_{k}^{*})\right)\;\;\;\; & = & \;\;\;\;\;\;\sum_{h=1}^{N}{\left[M^{*}\right]}_{j,h}\sum_{l=1}^{N}\;{\left[E\left(y_{n:1}y_{n:1}^{*}\right)\right]}_{(n-k)N+h,(n-k)N+l}{\left[M\right]}_{l,j}\\ \;\;\;\; & = & \;\;\;\;\;\;\;\sum_{h=1}^{N}{\left[M^{*}\right]}_{j,h}\;\sum_{l=1}^{N}\;\;{\left[E\left({\left(V_{n}\;⊗\;I_{N}\right)}^{*}x_{n:1}x_{n:1}^{*}\left(V_{n}\;⊗\;I_{N}\right)\right)\right]}_{(n-k)N+h,(n-k)N+l}\;{\left[M\right]}_{l,j}\\ \;\;\;\; & = & \;\;\;\;\;\;\;\sum_{h=1}^{N}{\left[M^{*}\right]}_{j,h}\;\sum_{l=1}^{N}\;\;{\left[{\left(V_{n}\;⊗\;I_{N}\right)}^{*}\;E\;\left(\;x_{n:1}x_{n:1}^{*}\;\right)\;\left(V_{n}\;⊗\;I_{N}\right)\right]}_{(n-k)N+h,(n-k)N+l}\;{\left[M\right]}_{l,j}\\ \;\;\;\; & = & \;\;\;\;\;\;\;\sum_{h=1}^{N}{\left[M^{*}\right]}_{j,h}\;\sum_{l=1}^{N}\;\;{\left[\;{\left(\;V_{n}\;⊗\;I_{N}\;\right)}^{*}\;U{\mathrm{diag}}_{1\leq p\leq nN}\;\;\left(\lambda_{p}\;\left(E\left(x_{n:1}x_{n:1}^{*}\right)\;\right)\;\right)\;\;\left(\;{\left(\;V_{n}\;⊗\;I_{N}\;\right)}^{*}\;U\;\right){\;}^{*}\right]}_{\;(n\;-\;k)\;N\;+\;h,(n\;-\;k)\;N\;+\;l\;}\;{\left[M\right]}_{l,j}\\ \;\;\;\; & = & \;\;\;\;\;\;\;\sum_{p=1}^{nN}\;\lambda_{p}\left(\;E\;\left(\;x_{n:1}x_{n:1}^{*}\;\right)\;\right){\left|\sum_{h=1}^{N}\bar{{\left[M\right]}_{h,j}}{\left[{\left(V_{n}\;⊗\;I_{N}\right)}^{*}U\right]}_{(n-k)N+h,p}\right|}^{2}, \end{array}$$

and thus,

$$\begin{array}{cc} \lambda_{nN}\left(E\left(x_{n:1}x_{n:1}^{*}\right)\right) & \sum_{p=1}^{nN}{\left|\sum_{h=1}^{N}\bar{{\left[M\right]}_{h,j}}{\left[{\left(V_{n}⊗I_{N}\right)}^{*}U\right]}_{(n-k)N+h,p}\right|}^{2}\\ \; & \leq \lambda_{j}\left(E(y_{k}y_{k}^{*})\right)\leq \lambda_{1}\left(E\left(x_{n:1}x_{n:1}^{*}\right)\right)\sum_{p=1}^{nN}{\left|\sum_{h=1}^{N}\bar{{\left[M\right]}_{h,j}}{\left[{\left(V_{n}⊗I_{N}\right)}^{*}U\right]}_{(n-k)N+h,p}\right|}^{2} \end{array}$$

for all j∈{1,…,N}. Hence, as

$$\begin{array}{cc} \sum_{p=1}^{nN} & {\left|\sum_{h=1}^{N}\;\bar{{\left[M\right]}_{h,j}}{\left[\;{\left(\;V_{n}\;⊗\;I_{N}\;\right)}^{*}\;U\right]}_{(n\;-\;k)N\;+\;h,p}\right|}^{2}\;\;=\;\;\sum_{h=1}^{N}\;{\left[M^{*}\right]}_{j,h}\;\sum_{l=1}^{N}{\left[\;{\left(\;V_{n}\;⊗\;I_{N}\;\right)}^{*}\;U{\left(\;{\left(\;V_{n}\;⊗\;I_{N}\;\right)}^{*}\;U\right)}^{*}\right]}_{(n\;-\;k)N\;+\;h,(n\;-\;k)N\;+\;l}\;{\left[M\right]}_{l,j}\\ \; & \;=\;\sum_{h=1}^{N}\;{\left[\;M^{*}\;\right]}_{j,h}\;\sum_{l=1}^{N}\;{\left[\;{\left(\;V_{n}\;⊗\;I_{N}\;\right)}^{*}\;I_{nN}\;\left(\;V_{n}\;⊗\;I_{N}\;\right)\;\right]}_{(n\;-\;k)N\;+\;h,(n\;-\;k)N\;+\;l}\;{\left[\;M\;\right]}_{l,j}\;=\;\sum_{h=1}^{N}\;{\left[\;M^{*}\;\right]}_{j,h}\;\sum_{l=1}^{N}\;{\left[\;I_{nN}\;\right]}_{(n\;-\;k)N\;+\;h,(n\;-\;k)N\;+\;l}\;{\left[\;M\;\right]}_{l,j}\;=\;1, \end{array}$$

Equation (3) holds. □

## Appendix C. Proof of Theorem 2

**Proof.** Fix $k\in \left\{1,\ldots ,n-1\right\}\setminus \left\{\frac{n}{2}\right\}$. Since

$$\begin{array}{cc} y_{k} & =\frac{1}{\sqrt{n}}\sum_{j=1}^{n}e^{-\frac{2\pi (j-1)k}{n}i}{\left[x_{n:1}\right]}_{j,1}=\frac{1}{\sqrt{n}}\sum_{j=1}^{n}\left(\cos\frac{2\pi (1-j)k}{n}+i\sin\frac{2\pi (1-j)k}{n}\right)x_{n-j+1}, \end{array}$$

we obtain

Eyk^yk^⊤=ERe(yk)Im(yk)Re(yk)⊤|Im(yk)⊤=ERe(yk)Re(yk)⊤ERe(yk)Im(yk)⊤EIm(yk)Re(yk)⊤EIm(yk)Im(yk)⊤=1n∑j1,j2=1ncos2π(1−j1)kncos2π(1−j2)knExn−j1+1xn−j2+1⊤cos2π(1−j1)knsin2π(1−j2)knExn−j1+1xn−j2+1⊤sin2π(1−j1)kncos2π(1−j2)knExn−j1+1xn−j2+1⊤sin2π(1−j1)knsin2π(1−j2)knExn−j1+1xn−j2+1⊤=1n∑j1,j2=1nAj1⊤Exn−j1+1xn−j2+1⊤Aj2,

where $A_{j}=\left(\;\;\begin{array}{ccc} \;\;\cos\frac{2\pi (1-j)k}{n}I_{N}\;\; & | & \;\;\sin\frac{2\pi (1-j)k}{n}I_{N}\;\; \end{array}\;\;\right)$ with j∈{1,…,n}. Fix r∈{1,…,2N}, and consider a real eigenvector v corresponding to $\lambda_{r}\left(E\left(\hat{y_{k}}{\hat{y_{k}}}^{⊤}\right)\right)$ with $v^{⊤}v=1$. Let $E\left(x_{n:1}x_{n:1}^{⊤}\right)=U{\mathrm{diag}}_{1\leq j\leq nN}\;\;\left(\lambda_{j}\left(E\left(x_{n:1}x_{n:1}^{⊤}\right)\right)\right)U^{-1}$ be an EVD of $E\left(x_{n:1}x_{n:1}^{⊤}\right)$, where *U* is real and orthogonal. Then

$$\begin{array}{cc} \lambda_{r}\left(E\left(\hat{y_{k}}{\hat{y_{k}}}^{⊤}\right)\right)\; & =\lambda_{r}\left(E\left(\hat{y_{k}}{\hat{y_{k}}}^{⊤}\right)\right)v^{⊤}v=v^{⊤}\lambda_{r}\left(E\left(\hat{y_{k}}{\hat{y_{k}}}^{⊤}\right)\right)v=v^{⊤}E\left(\hat{y_{k}}{\hat{y_{k}}}^{⊤}\right)v\\ \; & =\frac{1}{n}\sum_{j_{1},j_{2}=1}^{n}v^{⊤}A_{j_{1}}^{⊤}E\left(x_{n\;-\;j_{1}\;+\;1}x_{n\;-\;j_{2}\;+\;1}^{⊤}\right)A_{j_{2}}v=\frac{1}{n}\sum_{j_{1},j_{2}=1}^{n}v^{⊤}A_{j_{1}}^{⊤}{\left[E\left(x_{n:1}x_{n:1}^{⊤}\right)\right]}_{j_{1},j_{2}}A_{j_{2}}v\\ \; & =\frac{1}{n}\sum_{j_{1},j_{2}=1}^{n}v^{⊤}A_{j_{1}}^{⊤}e_{j_{1}}^{⊤}E\left(x_{n:1}x_{n:1}^{⊤}\right)e_{j_{2}}A_{j_{2}}v\\ \; & =\frac{1}{n}\sum_{j_{1},j_{2}=1}^{n}v^{⊤}A_{j_{1}}^{⊤}e_{j_{1}}^{⊤}U{\mathrm{diag}}_{1\leq p\leq nN}\;\;\left(\lambda_{p}\left(E\left(x_{n:1}x_{n:1}^{⊤}\right)\right)\right)U^{⊤}e_{j_{2}}A_{j_{2}}v\\ \; & =\frac{1}{n}\sum_{j_{1}=1}^{n}v^{⊤}A_{j_{1}}^{⊤}e_{j_{1}}^{⊤}U{\mathrm{diag}}_{1\leq p\leq nN}\;\;\left(\lambda_{p}\left(E\left(x_{n:1}x_{n:1}^{⊤}\right)\right)\right)\sum_{j_{2}=1}^{n}U^{⊤}e_{j_{2}}A_{j_{2}}v\\ \; & =\frac{1}{n}{\left[B^{⊤}{\mathrm{diag}}_{1\leq p\leq nN}\;\;\left(\lambda_{p}\left(\;E\;\left(x_{n:1}x_{n:1}^{⊤}\right)\;\right)\right)B\right]}_{1,1}\;=\;\frac{1}{n}\sum_{p=1}^{nN}\;{\left[B^{⊤}\right]}_{1,p}\lambda_{p}\left(\;E\;\left(x_{n:1}x_{n:1}^{⊤}\right)\;\right)\;{\left[B\right]}_{p,1}\\ \; & =\frac{1}{n}\sum_{p=1}^{nN}\lambda_{p}\left(E\;\left(x_{n:1}x_{n:1}^{⊤}\right)\;\right){\left[B\right]}_{p,1}^{2}, \end{array}$$

where $e_{l}\in C^{nN\times N}$ with ${\left[e_{l}\right]}_{j,1}=\delta_{j,l}I_{N}$ for all j,l∈{1,…,n} and $B=\sum_{j=1}^{n}U^{⊤}e_{j}A_{j}v$. Consequently,

$$\begin{array}{c} \lambda_{nN}\left(E\left(x_{n:1}x_{n:1}^{⊤}\right)\right)\frac{1}{n}\sum_{p=1}^{nN}{\left[B\right]}_{p,1}^{2}\leq \lambda_{r}\left(E\left(\hat{y_{k}}{\hat{y_{k}}}^{⊤}\right)\right)\leq \lambda_{1}\left(E\left(x_{n:1}x_{n:1}^{⊤}\right)\right)\frac{1}{n}\sum_{p=1}^{nN}{\left[B\right]}_{p,1}^{2}. \end{array}$$

Therefore, to finish the proof we only need to show that $\frac{1}{n}\sum_{p=1}^{nN}{\left[B\right]}_{p,1}^{2}=\frac{1}{2}$. Applying ([5] (Equations (14) and (15))) yields

1n∑p=1nNBp,12=1n∑p=1nNB⊤1,pBp,1=1nB⊤B=1n∑j1=1nU⊤ej1Aj1v⊤∑j2=1nU⊤ej2Aj2v=1n∑j1,j2=1nv⊤Aj1⊤ej1⊤ej2Aj2v=1n∑j=1nv⊤Aj⊤Ajv=1n∑j=1n(Ajv)⊤(Ajv)=1n∑j=1n∑s=1NAjvs,12=1n∑j=1n∑s=1Ncos2π(1−j)kn[v]s,1+sin2π(1−j)kn[v]N+s,12=1n∑s=1N∑j=1ncos2π(1−j)kn2[v]s,12+sin2π(1−j)kn2[v]N+s,12+2cos2π(1−j)knsin2π(1−j)kn[v]s,1[v]N+s,1=∑s=1N[v]s,121n∑j=1ncos2π(1−j)kn2+[v]N+s,121n∑j=1nsin2π(1−j)kn2+[v]s,1[v]N+s,11n∑j=1n2sin2π(1−j)kncos2π(1−j)kn=∑s=1N[v]s,121n∑j=1n1−sin2π(1−j)kn2+[v]N+s,122+[v]s,1[v]N+s,11n∑j=1nsin4π(1−j)kn=∑s=1N[v]s,121−1n∑j=1nsin2π(1−j)kn2+[v]N+s,122−[v]s,1[v]N+s,11n∑j=1nsin4π(j−1)kn=∑s=1N[v]s,122+[v]N+s,122−[v]s,1[v]N+s,11n∑j=1nIme4π(j−1)kni=∑s=1N[v]s,122+[v]N+s,122−[v]s,1[v]N+s,11nIm∑j=1ne4π(j−1)kni=∑s=1N[v]s,122+[v]N+s,122=12∑h=12N[v]h,12=12v⊤v=12.

 □

## Appendix D. Proof of Lemma 2

**Proof.** (1) $E\left(y_{k}y_{k}^{*}\right)={\left[E\left(y_{n:1}y_{n:1}^{*}\right)\right]}_{n-k+1,n-k+1}={\left[{\left(V_{n}\;⊗\;I_{N}\right)}^{*}E\left(x_{n:1}x_{n:1}^{*}\right)\left(V_{n}\;⊗\;I_{N}\right)\right]}_{n-k+1,n-k+1}$. (2) $E\left(y_{k}y_{k}^{⊤}\right)={\left[E\left(y_{n:1}y_{n:1}^{⊤}\right)\right]}_{n-k+1,n-k+1}={\left[{\left(V_{n}\;⊗\;I_{N}\right)}^{*}E\left(x_{n:1}x_{n:1}^{⊤}\right){\left({\left(V_{n}\;⊗\;I_{N}\right)}^{*}\right)}^{⊤}\right]}_{n-k+1,n-k+1}$. (3) We have

(A1) Eykyk*=ERe(yk)+iIm(yk)Re(yk)⊤−iIm(yk)⊤=ERe(yk)Re(yk)⊤+EIm(yk)Im(yk)⊤+iEIm(yk)Re(yk)⊤−ERe(yk)Im(yk)⊤,

and

(A2) Eykyk⊤=ERe(yk)+iIm(yk)Re(yk)⊤+iIm(yk)⊤=ERe(yk)Re(yk)⊤−EIm(yk)Im(yk)⊤+iEIm(yk)Re(yk)⊤+ERe(yk)Im(yk)⊤.

As

Eyk^yk^⊤=ERe(yk)Re(yk)⊤ERe(yk)Im(yk)⊤EIm(yk)Re(yk)⊤EIm(yk)Im(yk)⊤,

assertion (3) follows directly from Equations (A1) and (A2).  □
